# An exploration on COVID-19 vaccination motivation patterns from the perspective of the Chaxu culture in metropolis of China: A multi-center study

**DOI:** 10.3389/fpubh.2022.1065043

**Published:** 2022-12-22

**Authors:** Jiaoling Huang, Zhiyun Jiang, Jie Gu, Yuqi Yang, Yuge Yan, Xiaoqing Gu, Yundan Bai, Yan Liang

**Affiliations:** ^1^School of Public Health, Shanghai Jiao Tong University School of Medicine, Shanghai, China; ^2^Pudong Institute for Health Development, Shanghai, China; ^3^Department of General Practition, Zhongshan Hospital Fudan University, Shanghai, China; ^4^Zhongshan Hospital International Medical Center, Shanghai, China; ^5^College of Global Public Health, New York University, New York, NY, United States; ^6^Xidu Community Health Service Center of Fengxian District, Shanghai, China; ^7^Health Management Medical Center, Chengdu First People's Hospital, Chengdu, Sichuan Province, China; ^8^School of Nursing, Fudan University, Shanghai, China

**Keywords:** COVID-19 vaccination, motivation, Chaxu culture, SES, Chinese

## Abstract

**Objectives:**

Chaxugeju is a very special Chinese culture following a self-centered and outward expanding social network, which might be a significant culture factor for vaccination behavior. This study aimed to identify the motivation pattern in China, and paid special focus on socio-economic status (SES), region, and migration.

**Methods:**

We used a latent class analysis, with a sample of 12,432 participants collected in China from April to June, to identify the COVID-19 vaccination motivation patterns. Multinomial logistic regression models were utilized to separately explore associations between SES, migration, region, and COVID-19 vaccination motivation patterns.

**Results:**

Three COVID-19 vaccination motivation patterns were identified: Self-protection (41.9%), Trust and Self-protection (38.5%), and Trust and Differential Protection (19.6%). Participants with higher income were more likely to be Trust and Self-protection, and when income is more than 50,000 CNY per month, they are more likely to be self-protection. Professional/white collar were more likely to be Self-protection. Participants from Shenzhen were more likely to be Trust and Differential protection. The moderating effects of gender were found for income and region. Gender does not moderate the associations of occupation or migration and COVID-19 vaccination motivation patterns.

**Conclusion:**

Three motivational patterns were identified in which the Trust & Differential Pattern followed the traditional self-family-community Chaxu circle. However, the Chaxu motivation pattern was not the dominant one which might be weakened by SES. Migration and Shenzhen preserved the traditional social network, keeping in the trust and differential pattern. All of these factors in various cultural contexts should be considered when promoting vaccines.

## 1. Introduction

Countries across the world have experienced a tough period triggered by COVID-19 since the end of 2019, including medical system collapse, millions of deaths, financial crises, and impacts on daily life for billions of people (1, 2). We noticed that different strategies were adopted by different countries; however, vaccination was a common policy promoted by most countries (3, 4). Vaccination is regarded as an effective way to contain the spread of COVID-19, though the effectiveness against the new variants has declined according to current studies (5, 6).

Current studies have widely discussed COVID-19 vaccine hesitancy, covering disparities over age, occupation, and gender, and vaccine motivation as well (7–9). Most of the current motivation studies regarding the COVID-19 vaccine have examined the protection motivation theory or self-determination theory using path analyses or multiple regressions over different countries and populations (10–13). These studies have explored the key motivations of self and family member protection, trust of vaccine safety and efficacy, promoting/modeling vaccination, doing one's duty, being a helper, and ending the pandemic/restoring normalcy (14–16). Furthermore, key motivations of vaccine uptake were summarized into differentiated patterns, for instance, the pattern of infection-related risk perception, pandemic-related health concerns and vaccine-related safe protection (17, 18). However, region and culture factors have been scarcely touched.

We believe that there might be disparities in different culture contexts when considering vaccine motivation. For examples, China has long been deeply influenced by the Confucian culture. Chaxugeju, also called the differential mode of association, was proposed by Fei to reveal the interpersonal pattern of close and distant relationships (19). Unlike clearly defined social boundaries in western societies, the Chinese traditional society is “just like the circles that appear on the surface of a lake when a rock is thrown into it. Everyone stands at the center of the circles produced by his or her own” (20). Chaxugeju describes a relationship from me to close family members or relatives, and to more distant others, which is gradually transmitted from the near to the far, reflecting the very traditional Chinese interpersonal relationship, and also builds the basic traditional social structure. The differential pattern is also reflected in people's behavior patterns, including vaccination behavior, which is a significant cultural factor that cannot be ignored. Some studies have pointed out that the Chaxu culture has been gradually disintegrating with urbanization, and others have argued that the differential culture still has a profound impact on Chinese society, especially for rural-to-urban migrants (21). Urbanization changes are accompanied by some notable features, such as class differentiation related to socioeconomic factors, regional disparities of urban-rural and west-east, and migrants' social roles, all of which have not been considered in the existing motivation studies in vaccination. Thus, we paid special attention of SES, migration, and region factors in the context of Chaxu culture in this vaccine motivation study. The extent to which their relation with vaccine motivation is identical, is unclear. We assumed that SES, migration and region may not represent the same causal processes, so we studied the relations between education, income, occupation, migration, and region (seen as five distinct social dimensions) and COVID-19 vaccination motivation pattern.

Metropolis were hardest hit by waves of COVID-19, with high population density and massive population movement. Thus, we paid special attention in vaccination motivation patterns in metropolitan areas in China, examining whether citizens' motivation follows the traditional Chaxu culture, in which region, SES, and migration were considered as significant factors.

## 2. Methods

### 2.1. Study design, setting, and participants

This was a multi-center, cross-sectional survey conducted in three metropolitan areas: Chengdu (the capital city of Sichuan Province in western China), Shanghai (in eastern China), and Shenzhen (in southeastern China). The survey included adults who were vaccinated against COVID-19 in China between April 1, 2021 and June 1, 2021. We used a self-administered questionnaire to collect data. Trained nurses and general practitioners performed the data collection in community health service centers. The online questionnaire and informed consent forms were provided to participants through a link, and a printed version was provided if the online version was not accessible.

A multi-stage, stratified, systematic sampling method was used. First, the cities were selected based on China's city-tier classification, with Shanghai and Shenzhen as Tier 1 and Chengdu as emerging Tier 1. Although both Shanghai and Shenzhen are the most developed cities, some differences exist. For example, compared with Shanghai, the age demographic of Shenzhen is on the younger, and Shenzhen has more rural-urban migrants; Shanghai has more migrants than Shenzhen, while Shenzhen has the highest migrant proportion in China's first-tier cities. Second, three representative districts (downtown, sub-urban, and rural) were selected from each city (a total of nine districts or counties). Third, two community health service centers (CHSCs) were randomly selected from each district/county (a total of 18 CHSCs). Fourth, we recruited individuals whose vaccination ID ended with eight during the post-vaccination observation period. The inclusion criteria were as follows: (1) age equal to or >18 years; and (2) the ability to communicate, grant consent, and participate. The exclusion criteria were as follows: (1) pregnant or lactating women; and (2) inability to communicate with interviewers or to grant informed consent. The minimum sample size for each city was calculated for statistical significance. The sample size calculation was reported in previous research (22) and the required sample size was estimated from the estimated proportion of individuals who were highly hesitant to vaccination. Altogether, a total of 17,656 individuals were recruited, and 17,560 individuals completed the survey. Finally, as we focused on the labor age population in this study, we excluded potential college students and retired older adults to maintain the homogeneity of the labor age population, and we included adults aged between 25 and 54 years as the analysis sample (*N* = 12,432).

### 2.2. Measures

#### 2.2.1. Dependent variable

The COVID-19 vaccination motivation pattern was the dependent variable in this study. We used multiple choice questions to capture participants' COVID-19 vaccination motivations: what are your COVID-19 vaccination motivations? Eleven possible responses (see Additional File 1) were developed and provided based on a comprehensive literature review of similar topics (15, 23–25). We coded each response as a dichotomous variable. A total of 11 items were used in the latent class analysis (LCA) model to identify the COVID-19 vaccination motivation patterns (Flow diagram of the selection of eligible participants is provided in Supplementary material 1).

#### 2.2.2. Focal independent variables

Education, income, and occupation were chosen as indicators of SES. Education was measured as a categorical variable: illiteracy and primary school, junior high school, senior high school, university and junior college, and master's degree and above. Income was also measured as a categorical variable based on the self-reported monthly household income (CNY): <5,000, ≥5,000 and <10,000, ≤ 10,000 and <20,000, ≥20,000 and <50,000, and ≥50,000. Occupation was dichotomized as manual/unskilled service or professional/white collar. Migration was defined as without registered residence (huji) in the current city. Region was used as an individual level measurement.

#### 2.2.3. Other measures

Several covariates were included in the analysis as potential co-founders: (1) individual factors, including age, gender, self-rated health, chronic disease, disability, health behavior (including smoking, drinking, and doing exercise), and knowledge about vaccines; (2) family factors, including marital status, living status, family relation; and (3) social factors, defined as perceptions of social environmental safety, including an evaluation of the government's prevention and control measures and outcomes, and concerns regarding international outbreak spread. Details on the covariates are provided in the Additional File 1.

### 2.3. Data analysis

Descriptive statistics were used to summarize participant characteristics. We used the LCA to identify the COVID-19 vaccination motivation patterns among Chinese adults. A series of LCA models ranging from 2 to 5 classes were adopted for the 12,432 adults aged between 25 and 54 years to determine latent classes in the COVID-19 vaccination motivation patterns. The optimal model that combined goodness of fit and parsimony was selected based on various statistical fit indices and interpretability (26). Statistical indices reported here include (27): the Akaike Information Criterion (AIC); Bayesian Information Criterion (BIC); sample-size adjusted BIC (aBIC); Lo–Mendell–Rubin likelihood ratio test (LMR); bootstrap likelihood ratio test (BLRT); and an entropy measure.

Multivariable multinomial logistic regression analyses were performed to calculate the effects of education, income, occupation, migration, and region before and after mutual adjustments. We studied the relations between education, income, occupation, migration, and region (seen as five distinct social dimensions) and the outcome (motivation pattern). Multivariable multinomial logistic regression models were used to separately determine the associations between the focal independent variables (education, income, occupation, migration, and region) and the COVID-19 vaccination motivation patterns. A series of multinomial logistic regression models were adopted to control for the confounders, only individual factors, both individual and family factors, and all individual, family, and social factors. We refer to these as “gross effects” below. Additionally, the effects are estimated simultaneously to examine whether each social dimension (education, income, occupation, migration, and region) shows an effect when the other focal independent variables are adjusted. We refer to these as “net effects”, or “effects after mutual adjustment”. In addition, we tested an interaction between gender and the focal independent variable (SES, migration, and region). LCA models were conducted in Mplus version 8.0 and all subsequent analyses were performed using Stata SE version 17 (StataCorp LLC, College Station, TX).

## 3. Results

### 3.1. Participant characteristics

Table 1 shows the participants' characteristics in the overall sample and in men and women. The sample consisted of 56.4% men; a total of 53.9% of the participants were aged between 25 and 34 years, with 31.5% aged between 35 and 44 years; and 62.8% were married. Table 1 also shows the SES, migration, and region of the participants. The majority (40.3%) of the participants attained university and junior college education, and 28.6% of the participants had an income between 5,000 and 10,000 CNY per month, followed by those with income <5,000 CNY per month (27.6%) and those with income between 10,000 and 20,000 CNY per month (17.4%). The majority of the participants (57.2%) were classified as having professional/white collar occupations. The majority (59.9%) of the participants were migrants. In terms of region, 35.9% were from Chengdu, followed by those from Shanghai (32.4%) and Shenzhen (31.7%).

**Table 1:** Characteristics of the sample included in this study (*n* = 12,432).

**Characteristics**	**Full sample**	**Gender-stratified sample**	
		**Male**	**Female**
	***n*** **(%) or M ±SD**	***n*** **(%) or M ±SD**	***n*** **(%) or M ±SD**
**Age group**			
25–34	6,701 (53.9)	3,933 (56.1)	2,768 (51.0)
35–44	3,912 (31.5)	2,098 (29.9)	1,814 (33.5)
45–54	1,819 (14.6)	979 (14.0)	840 (15.5)
**Marital status**			
Single	3,847 (30.9)	2,725 (38.9)	1,122 (20.7)
Married	7,805 (62.8)	3,955 (56.4)	3,850 (71.0)
Divorced	527 (4.2)	242 (3.4)	285 (5.3)
Widowed	253 (2.1)	88 (1.3)	165 (3.0)
**Education**			
Illiteracy and primary school	251 (2.0)	96 (1.4)	155 (2.9)
Junior high school	3,050 (24.5)	1,584 (22.6)	1,466 (27.0)
Senior high school	3,081 (24.8)	2,032 (29.0)	1,049 (19.3)
University and junior college	5,012 (40.3)	2,698 (38.5)	2,314 (42.7)
Master and above	1,038 (8.4)	600 (8.5)	438 (8.1)
**Income (monthly**, ¥**)**			
< 5,000	3,427 (27.6)	1,865 (26.6)	1,562 (28.8)
≥5,000 and < 10,000	3,555 (28.6)	2,171 (31.0)	1,384 (25.5)
≥10,000 and < 20,000	2,169 (17.4)	1,219 (17.4)	950 (17.5)
≥20,000 and < 50,000	1,595 (12.8)	832 (11.9)	763 (14.1)
≥50,000	1,686 (13.6)	923 (13.1)	763 (14.1)
**Occupation**			
Manual/unskilled service	5,321 (42.8)	3,033 (43.3)	2,288 (42.2)
Professional/white collar	7,111 (57.2)	3,977 (56.7)	3,134 (57.8)
**Migration**			
Non-migrant	4,988 (40.1)	2,353 (33.6)	2,635 (48.6)
Migrant	7,444 (59.9)	4,657 (66.4)	2,787 (51.4)
**Region**			
Chengdu	4,465 (35.9)	2,188 (31.2)	2,277 (42.0)
Shanghai	4,031 (32.4)	2,092 (29.9)	1,939 (35.8)
Shenzhen	3,936 (31.7)	2,730 (38.9)	1,206 (22.2)

### 3.2. Identification of the COVID-19 vaccination motivation patterns

The results of the LCA are presented in Table 2. Four latent class models were created, specifying latent class counts from 2 to 5. According to recommendations of the model selection in the LCA, we chose a three-class solution as the best fitting model. In a large sample, the Akaike information criterion (AIC), the Bayesian information criterion (BIC), and the sample-size adjusted BIC (aBIC) will continue to decrease with an increase in the latent class counts. However, the three-class model showed the highest entropy (0.913), representing the highest certainty of classification. Moreover, the three-class model was interpretable and reasonably well-defined (Table 3). Table 3 shows the three-class model that was the final solution of COVID-19 vaccination motivation patterns.

**Table 2:** LCA model fit statistics.

**Classes**	**AIC**	**BIC**	**aBIC**	**Entropy**	**LMR**	**BLRT**	**Relative class size**
2	129358.653	129529.498	129456.406	0.879	< 0.0001	< 0.0001	43.3/56.7
3	119508.020	119768.001	119656.774	0.913	< 0.0001	< 0.0001	19.6/38.5/41.9
4	117223.779	117572.896	117423.535	0.871	< 0.0001	< 0.0001	19.7/11.9/37.2/31.2
5	115970.373	116408.627	116221.131	0.828	< 0.0001	< 0.0001	17.6/23.0/16.0/30.4/13.0


Note: AIC, Akaike information criterion; BIC, Bayesian information criterion; aBIC, sample-size adjusted BIC; LMR, p-value for the Lo-Mendell-Rubin likelihood ratio test; BLRT, p-value for the bootstrapped likelihood ratio test.

**Table 3:** Proportion of participants within each latent class assignment based on motivations (*n* = 12,432).

**Motivations**	**Trust and differential protection**	**Trust and self-protection**	**Self-protection**
Trust in the safety of vaccines	100	94.8	6.4
Trust in the effectiveness of vaccines	100	91.0	3.1
Self-protection	99.7	98.1	85.1
Protection of family members	99.3	87.7	49.7
Protection of neighbors, friends and colleagues	98.0	54.2	15.2
Contributing to herd immunity	99.9	75.7	27.8
Perceived personal obligations influenced by media publicity	98.2	31.8	7.0
People around have vaccinated	90.1	12.9	6.6
Vaccinated people around me did not experience side effects	93.2	25.4	3.5
Company-based mobilization	89.5	37.0	27.2
Community mobilization	88.3	19.4	4.4
Percentage of cohort	19.6	38.5	41.9

The final latent classes were as follows: (1) participants who presented trust in the safety and effectiveness of vaccines, protection motivation for self, family members, neighbors, friends, and colleagues, and the contribution to herd immunity (19.6% of participants, “Trust and Differential Protection”); (2) participants who presented trust in the safety and effectiveness of vaccines and self- protection motivation (38.5% of participants, “Trust and Self-protection”); and (3) participants who presented self-protection motivation (41.9% of participants, “Self-protection”). Table 2 presents the proportion of the 12,432 participants within each latent class assignment having each COVID-19 vaccination motivation category.

### 3.3. Multinominal logistic regression analyses for the COVID-19 vaccination motivation patterns

Table 4 presents the results of multinominal logistic regression analyses (”gross effects”). Detailed results on covariates are provided in Supplementary materials 2–6.

**Table 4:** Motivation patterns multinominal logistic regression (*n* = 12,432).

**Variable**	**Model 1A-1E**		**Model 2A-2E**		**Model 3A-3E**	
	**Motivation patterns**		**Motivation patterns**		**Motivation patterns**	
	**(Trust and differential protection)1**		**(Trust and differential protection)**		**(Trust and differential protection)**	
	**Trust and** **self-protection2** **OR (95% CI)**	**Self-protection** **OR (95% CI)3**	**Trust and** **Self-protection** **OR (95% CI)**	**Self-protection** **OR (95% CI)**	**Trust and Self-protection** **OR (95% CI)**	**Self-protection** **OR (95% CI)**
**Panel A: Independent variable: education**						
Education (ref: illiteracy and primary school)						
Junior high school	1.89^**^(1.20–2.97)	0.66^*^(0.45–0.96)	1.89^**^(1.20–2.98)	0.68^*^(0.46–1.00)	1.88^**^(1.20–2.96)	0.69 (0.47–1.02)
Senior high school	1.82^*^(1.16–2.86)	0.53^**^(0.36–0.78)	1.83^**^(1.16–2.88)	0.55^**^(0.37–0.81)	1.82^*^ (1.16–2.87)	0.59^**^(0.40–0.87)
University and junior college	1.92^**^(1.22–3.02)	0.64^*^(0.43–0.94)	1.91^**^(1.21–3.00)	0.68^*^(0.46–1.00)	1.91^**^(1.21–3.01)	0.76 (0.52–1.13)
Master and above	2.16^**^(1.33–3.49)	0.90(0.59–1.36)	2.17^**^(1.33–3.52)	0.95(0.62–1.45)	2.17^**^(1.34–3.53)	1.12 (0.73–1.71)
**Panel B: Independent variable: income**						
Income (ref: < 5,000, monthly, ¥)						
≥5,000 and < 10,000	1.18^*^(1.04–1.35)	1.00 (0.94–1.05)	1.17^*^(1.03–1.34)	1.06 (0.93–1.21)	1.18^*^(1.03–1.35)	1.12 (0.98–1.28)
≥10,000 and < 20,000	1.20^*^(1.03–1.39)	1.07 (0.92–1.25)	1.17^*^(1.01–1.37)	1.10 (0.95–1.28)	1.18^*^(1.01–1.37)	1.20^*^(1.03–1.4)
≥20,000 and < 50,000	1.21^*^(1.02–1.43)	1.12 (0.94–1.32)	1.19^*^(1.00–1.41)	1.14 (0.97–1.35)	1.19^*^(1.00–1.41)	1.23^*^(1.04–1.46)
≥50,000	1.21^*^(1.03–1.43)	1.14 (0.96–1.34)	1.20^*^(1.02–1.42)	1.19^*^(1.01–1.41)	1.21^*^(1.02–1.43)	1.32^**^(1.11–1.56)
**Panel C: Independent variable: occupation**						
Occupation (ref: manual/unskilled service)						
Professional/white collar	1.01 (0.91–1.12)	1.05 (0.95–1.16)	1.00 (0.90–1.11)	1.06 (0.96–1.17)	1.00 (0.91–1.11)	1.12^*^(1.02–1.25)
**Panel D: Independent variable: migration**						
Migration (ref: non-migrant)						
Migrant	0.96 (0.86–1.06)	0.88^*^(0.79–0.98)	0.99 (0.88–1.10)	0.85^**^(0.76–0.95)	0.98 (0.88–1.10)	0.82^**^(0.74–0.92)
**Panel E: Independent variable: region**						
Region (ref: Chengdu)						
Shanghai	1.03 (0.91–1.17)	1.32^***^(1.17–1.5)	1.04 (0.91–1.17)	1.32^***^(1.16–1.49)	1.04 (0.91–1.18)	1.35^***^(1.19–1.53)
Shenzhen	0.79^***^(0.70–0.89)	0.78^***^(0.69–0.88)	0.80^***^(0.70–0.90)	0.72^***^(0.64–0.82)	0.79^***^(0.70–0.89)	0.66^***^(0.58–0.75)

Notes:

* p < 0.05;

** p < 0.01;

*** p < 0.001.

Model 1A-1E: association between education (income/occupation/migration/region) and motivation patterns, adjusted for age, gender, self-rated health, health behavior, and knowledge about vaccines (individual factors).

Model 2A-2E: association between education (income/occupation/migration/region) and motivation patterns, adjusted for age, gender, self-rated health, health behavior, and knowledge about vaccines, marital status, living arrangements, and family relation (individual and family factors).

Model 3A-3E: association between education (income/occupation/migration/region) and motivation patterns, adjusted for age, gender, self-rated health, health behavior, and knowledge about vaccines, marital status, living arrangements, family relation, and perceptions of environmental safety (individual, family and social factors).

Sensitivity analyses: we also conducted additional analyses to assess the robustness of results, by including education, income, and occupation in the same model. These inferences were qualitatively similar (Supplementary material 14).

#### 3.3.1. Trust and self-protection vs. trust and differential protection

Participants with higher education levels were more likely to be in the Trust and Self-protection class than in the Trust and Differential Protection class. A similar trend was observed for income. No significant results were found for occupation and migrant. In terms of region, participants from Shenzhen were less likely to be in the Trust and Self-protection class than in the Trust and Differential Protection class. These results were observed when controlling for only the individual factors, both individual and family factors, as well as all individual, family, and social factors.

#### 3.3.2. Self-protection vs. trust and differential protection

Participants with higher education levels (junior high school, senior high school, or university and junior college) were less likely to be in the Self-protection class than in the Trust and Differential Protection class when controlling for only individual factors or both individual and family factors. After controlling for all individual, family, and social factors, the trend was only observed for senior high school (Odds ratio 0.59, 95% CI: 0.40–0.87). When controlling for both the individual and family factors, participants with income >50,000 CNY per month were more likely to be in the Self-protection class than in the Trust and Differential Protection class. A similar trend was observed for participants with income >10,000 CNY per month (≥10,000 and <20,000, ≥20,000 and <50,000, or ≥50,000) when all individual, family, and social factors were controlled for. Participants who were into professional/white collar occupations were more likely to be in the Self-protection class than in the Trust and Differential Protection class when all individual, family, and social factors were controlled for.

Migrants were less likely to be in the Self-protection class than in the Trust and Differential Protection class. Participants from Shanghai were more likely to be in the Self-protection class than in the Trust and Differential Protection class. Participants from Shenzhen were less likely to be in the Self-protection class than in the Trust and Differential Protection class. These results were observed when controlling for only individual factors, both individual and family factors, as well as for all individual, family, and social factors. After mutual adjustment, education, income, and region all showed some significance in all models, although the magnitude varies (Supplemental material 7). However, occupation and migration showed no significance in simultaneous estimation the (net) effects, although the significance existed for separate estimation (gross effects).

### 3.4. Moderating effects of gender on the COVID-19 vaccination motivation patterns

Gender moderated the associations of education and the COVID-19 vaccination motivation patterns (Supplementary material 8). Detailed results on covariates are provided in Supplementary materials 9–13. The moderating effects of gender were also found for income and region. Gender did not moderate the associations of occupation or migration and the COVID-19 vaccination motivation patterns. Women who had master degrees and above were more likely to have the Trust and Differential Protection Pattern rather than the Trust and Self-protection Pattern. Women who had income of >50,000 CNY per month were more likely to have the self-protection Pattern rather than the Trust and Differential Protection Pattern. Women from Shanghai were more likely to have the Self-protection Pattern rather than the Trust and Differential Protection Pattern. These results were observed when controlling for only individual factors, both individual and family factors, as well as all individual, family, and social factors.

## 4. Discussion

Interestingly, three motivation patterns of COVID-19 vaccination were found based on the LCA model that were named Self-protection, Trust and Self-protection, and Trust and Differential Protection. Consistent with the current studies, protection and trust were the primary components of vaccination motivation (25). In the context of China, we observed that the Trust and Differential Protection Pattern followed the traditional Chaxu cultural attitudes, with an apparent self-family-community protection circle in which reflecting a traditional me-to-others relationship. In addition, Self-protection Motivation Patterns with or even without trust were also identified, and the latter was self-protection motivated. However, a lack of basic trust in vaccine safety and efficacy seemed to be a self-contradictory state. We concluded that this could have been due to the initial stage of vaccination promotion that caused a lack of trust, and such concerns were also found in the research conducted in other settings (23). Such a distinctive phenomenon found in the metropolitan areas of China was surprisingly consistent with the western sociology theory, i.e., the social capital theory, which is a broad theoretical construct that explains the social resource sharing mechanism with some combination of distinct yet related contextual dimensions of trust, social networks, and norms of reciprocity (28). In the setting of vaccine motivation in China, protection is a situational expression of a reciprocal norm based on social networks.

We found that the Chaxu motivation pattern was not the predominant one in the metropolitan areas in China that might be disintegrated by rapid urbanization and class differentiation. Models showed that populations with higher income or non-manual labor occupations had higher probabilities distributed in Self-protection or Trust and Self-protection, rather than a self-family-community protection pattern. The traditional society relies on close social network relations to operate, but studies have shown that urbanization can disrupt existing social structures and exacerbate social stratification, and this dilutes connections between people (29). For example, traditional society is often confined to small communities, and people are familiar with each other. However, in the modern society, people are alienated and isolated from the community they live in. Higher-class groups are less dependent on traditional social network relationships, Chaxugeju, but tend to have a Self-protection Pattern. However, education, one of the SES measurements, worked on the motivation pattern in the opposite way. Survey participants with higher education levels tended to get vaccinated for the trust and differential-protection pattern. Current studies have demonstrated that education can help improve social trust (30); however, diversity of social capital can erode social trust and participation, and the latter was a prominent line of scholarship globally that linked ethno-racial diversity to undesirable collective outcomes, most notably, low levels of trust, civic engagement, and social capital (31). A causal conclusion could not be made in this study, but we obtained a preliminary outline where education, income, and occupation were all SES measurement variables, but the latter two, as important measurement variables of social capital, played a negative role in social trust, while the former played a positive role. Based on the fundamental disparities, the three had diametrically opposite roles in the motivation mechanism.

However, the Chaxu culture was also preserved in vaccine motivation to some degree. Compared with the Self-protection motivation pattern, migrants tended to follow a differential and trust pattern. Migrants are products of rapid urbanization in China. Chinese internal migration is a unique event in human history and may well be one of the greatest migration events ever to have taken place (32). Economic reforms since 1978 have brought about an unprecedented surge in China's migrant population. According to the National Bureau of Statistics, the size of the migrant population was 380 million in 2020, accounting for over 26.9% of the national total population (33). The majority of migrants are rural-urban individuals who have worked and lived in cities for years but are still officially registered as residents in their rural places of origin and are thus viewed as a “floating population.” (34). Although the huge migrant population used to provide cheap and flexible labor forces that have fostered industrialization and urbanization in China's post-reform development, their floating or unstable status in the spatial and social senses has been increasingly regarded as unsustainable in the future (35). Current studies have demonstrated that migrants rely more on social networks compared with local residents to integrate into new living environments or seek non-agriculture jobs (offering information, providing financial aid, or recommending a job) (36). Similarly, our study also revealed that participants from Shenzhen, rather than Shanghai, also tended to have the trust and differential pattern for vaccination. With more rural-urban migrants in Shenzhen, more traditional culture was observed in Shenzhen. It seems that Shenzhen was more influenced by Chaxugeju than Shanghai. A social network study in Shenzhen found that after migration, migrants rebuild their social networks along with changes in their occupations and surroundings (37). Thus, it is not surprising that the self-centered social network circle was also captured in the vaccination motivation in the group of migrants and in Shenzhen as well.

Our study also showed that motivation patterns varied by gender that acted as a moderator in the motivation pattern. Gender differences in motivation were widely discussed, covering education, occupation, migration, and healthcare service utilization. Specifically, in this study, females with higher income were likely to get vaccinated for Self-protection, and those with higher education levels were prone to differential protection when obtaining the vaccination, however, such trends were not found in male groups. Compared with males, females donate more money to charities and volunteer in greater numbers; men and women differ in their motivations for volunteering. Education was shown to be positively correlated with female participation (38, 39). Thus, females with higher education levels behaved in a more traditional way, with self-family-community protection and a different internal function mechanism.

Vaccination promotion is a worldwide campaign to fight against mutated viruses of COVID-19, and we observed the efforts made by health departments of countries across the world. Based on our research findings, we propose that cultural factors should be considered when continually advancing vaccination efforts. People in metropolitan areas of China have subverted the previous self-centered circle theory and are more concerned about the safety and effectiveness of vaccines and the safety of individuals. In other context, similar vaccine motivation was found that the most commonly reported reasons to get vaccinated were to protect self, family and others (40). Even so, in countries with contexts and cultures different from that in China, we believe more culture context should be taken into consideration. Heidi Larson, whose recent book, Stuck, summarized her decades of research on vaccine hesitancy, pointed out that vaccine reluctance and refusal are not issues that can be addressed by delivering more or better information, but issues involving broader social factors including history, region, and beliefs (41). We believe that vaccine trust is the primary condition for vaccination, and on this basis, different patterns of behavioral motivation differences are derived. These patterns included protection of individuals and families, empathy, and the restoration of regional social order, all of which should be seriously considered by local authorities, as Larson proposed, before you attempt to persuade, try to understand.

This study still has some limitations. First, although it was a multi-center study, comparisons between urban and rural areas are lacking. There are significant disparities in China, including economic development, social structures, and cultural customs. It is worth comparing vaccine motivation patterns in urban and rural China in further research. Second, a longitudinal analysis is necessary to track the changes in motivation over time.

## Data availability statement

The original contributions presented in the study are included in the article/Supplementary material, further inquiries can be directed to the corresponding author.

## Ethics statement

The studies involving human participants were reviewed and approved by Fengxian District Central Hospital Medical Ethic Committee. The patients/participants provided their written informed consent to participate in this study.

## Author contributions

JH and YL designed and conceived the study, were in charge of acquisition, analysis, or interpretation of data, and drafted the manuscript. YL and JG were responsible for data integrity and data analysis accuracy. ZJ conducted critical revisions of the important intellectual content. JG and JH performed the analyses and reviewed the data. JH obtained funding. YYang, YYan, YB, and XG provided administrative, technical, and material support. YL provided supervision. All authors reviewed and approved final version of the manuscript.
