# Supplementary material for: An exploration on COVID-19 vaccination motivation patterns from the perspective of the Chaxu culture in metropolis of China: A multi-center study
Source: Front Public Health. 2022 Dec 22;10:1065043. doi: 10.3389/fpubh.2022.1065043 (PMC9815457; doi:10.3389/fpubh.2022.1065043)
Supplement: Supplementary file 1 [file Data_Sheet_1.docx]

**
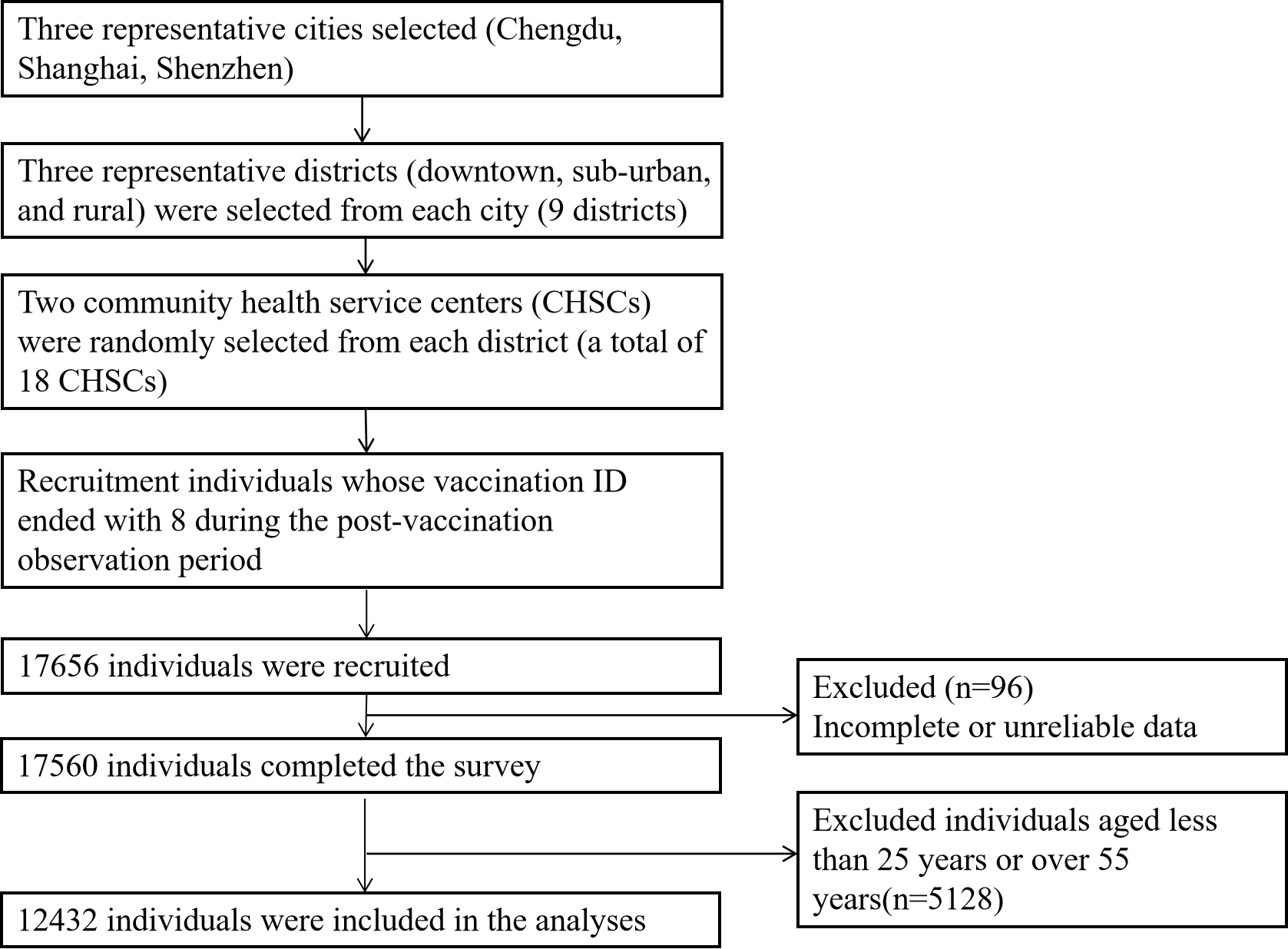
**

**S1 Figure.** Flow chart.

S2 Table. Replication of Table 4 presenting detailed results (Model 1A-3A) on covariates.

| Variable | Model 1A | | Model 2A | | Model 3A | |
| --- | --- | --- | --- | --- | --- | --- |
|  | Motivation patterns (Trust & differential protection) | | Motivation patterns (Trust & differential protection) | | Motivation patterns (Trust & differential protection) | |
|  | Trust & self-protection  OR (95% CI) | Self-protection  OR (95% CI) | Trust & self-protection  OR (95% CI) | Self-protection  OR (95% CI) | Trust & self-protection  OR (95% CI) | Self-protection  OR (95% CI) |
| Education (ref: illiteracy and primary school) |  |  |  |  |  |  |
| Junior high school | 1.89**(1.20−2.97) | 0.66* (0.45−0.96) | 1.89**(1.20−2.98) | 0.68* (0.46−1.00) | 1.88**(1.20−2.96) | 0.69 (0.47−1.02) |
| Senior high school | 1.82* (1.16−2.86) | 0.53**(0.36−0.78) | 1.83**(1.16−2.88) | 0.55**(0.37−0.81) | 1.82* (1.16−2.87) | 0.59**(0.40−0.87) |
| University and junior college | 1.92**(1.22−3.02) | 0.64* (0.43−0.94) | 1.91**(1.21−3.00) | 0.68* (0.46−1.00) | 1.91**(1.21−3.01) | 0.76 (0.52−1.13) |
| Master and above | 2.16**(1.33−3.49) | 0.90 (0.59−1.36) | 2.17**(1.33−3.52) | 0.95 (0.62−1.45) | 2.17**(1.34−3.53) | 1.12 (0.73−1.71) |
| Age group (ref: 25 – 34 years) |  |  |  |  |  |  |
| 35 − 44 | 1.52***(1.36−1.70) | 1.36***(1.21−1.52) | 1.47***(1.30−1.65) | 1.42 (1.26−1.61) | 1.47***(1.30−1.65) | 1.45***(1.29−1.64) |
| 45 − 54 | 2.04***(1.73−2.42) | 2.21***(1.87−2.61) | 1.96***(1.64−2.34) | 2.35 (1.97−2.80) | 1.97***(1.65−2.36) | 2.52***(2.11−3.01) |
| Gender (ref: male) |  |  |  |  |  |  |
| Female | 1.35***(1.20−1.53) | 1.12 (0.99−1.26) | 1.34***(1.18−1.51) | 1.15* (1.02−1.30) | 1.34***(1.18−1.51) | 1.15* (1.02−1.30) |
| Self-rated health | 0.96 (0.90−1.04) | 0.87***(0.81−0.93) | 0.98 (0.91−1.06) | 0.91* (0.85−0.98) | 0.98 (0.91−1.06) | 0.93 (0.86−1.00) |
| Chronic disease (Yes) | 0.97 (0.86−1.10) | 0.96 (0.84−1.08) | 0.98 (0.86−1.11) | 0.96 (0.84−1.08) | 0.98 (0.86−1.11) | 0.91 (0.80−1.03) |
| Disability (Yes) | 0.92 (0.58−1.44) | 0.82 (0.53−1.28) | 0.89 (0.56−1.39) | 0.75 (0.48−1.18) | 0.87 (0.55−1.37) | 0.74 (0.47−1.16) |
| Health behavior |  |  |  |  |  |  |
| Smoking | 0.97 (0.93−1.01) | 0.95**(0.91−0.98) | 0.97 (0.93−1.01) | 0.95* (0.91−0.99) | 0.97 (0.93−1.01) | 0.95* (0.92−0.99) |
| Drinking | 1.02 (0.97−1.08) | 0.99 (0.94−1.04) | 1.03 (0.97−1.08) | 0.99 (0.94−1.04) | 1.03 (0.97−1.08) | 0.99 (0.94−1.04) |
| Exercising | 1.06* (1.00−1.12) | 0.99 (0.94−1.05) | 1.06* (1.01−1.12) | 1.00 (0.95−1.05) | 1.06* (1.01−1.12) | 1.00 (0.95−1.06) |
| Knowledge about vaccines | 1.00 (0.95−1.06) | 0.75***(0.71−0.79) | 1.00 (0.95−1.06) | 0.76***(0.72−0.80) | 1.01 (0.95−1.07) | 0.79***(0.75−0.84) |
| Marital status (ref: single) |  |  |  |  |  |  |
| Married |  |  | 1.04 (0.91−1.19) | 0.89 (0.78−1.02) | 1.04 (0.91−1.19) | 0.88 (0.76−1.01) |
| Divorced |  |  | 0.85 (0.65−1.10) | 0.69**(0.53−0.89) | 0.85 (0.66−1.11) | 0.71* (0.54−0.92) |
| Widowed |  |  | 0.68* (0.48−0.97) | 0.63**(0.44−0.88) | 0.68* (0.48−0.97) | 0.59**(0.42−0.84) |
| Living arrangements (ref: living alone) |  |  |  |  |  |  |
| Living with family members |  |  | 1.17* (1.01−1.35) | 1.02 (0.88−1.17) | 1.17* (1.01−1.36) | 1.06 (0.92−1.22) |
| Co-rental |  |  | 1.04 (0.86−1.26) | 0.95 (0.79−1.14) | 1.04 (0.86−1.26) | 0.95 (0.79−1.15) |
| Else |  |  | 1.17 (0.95−1.45) | 0.97 (0.79−1.20) | 1.17 (0.95−1.45) | 0.96 (0.78−1.19) |
| Family relation |  |  | 0.94 (0.89−1.00) | 0.83***(0.78−0.88) | 0.95 (0.89−1.01) | 0.87***(0.82−0.92) |
| Perceptions of environmental safety |  |  |  |  |  |  |
| Government's prevention and control measures |  |  |  |  | 0.97 (0.90−1.05) | 0.88**(0.81−0.95) |
| Government's prevention and control outcomes |  |  |  |  | 0.98 (0.90−1.08) | 0.72***(0.66−0.79) |
| Concerns about the international outbreak spread |  |  |  |  | 1.00 (0.96−1.04) | 1.09***(1.05−1.13) |

Notes: **p* < 0.05; ***p* < 0.01; ****p* < 0.001

Model 1A: association between education and motivation patterns, adjusted for age, gender, self-rated health, chronic disease, disability, health behavior, and knowledge about vaccines (individual factors);

Model 2A: association between education and motivation patterns, adjusted for age, gender, self-rated health, chronic disease, disability, health behavior, and knowledge about vaccines, marital status, living arrangements, and family relation (individual and family factors);

Model 3A: association between education and motivation patterns, adjusted for age, gender, self-rated health, chronic disease, disability, health behavior, and knowledge about vaccines, marital status, living arrangements, family relation, and perceptions of environmental safety (individual, family and social factors);

S3 Table. Replication of Table 4 presenting detailed results (Model 1B-3B) on covariates.

| Variable | Model 1B | | Model 2B | | Model 3B | |
| --- | --- | --- | --- | --- | --- | --- |
|  | Motivation patterns (Trust & differential protection) | | Motivation patterns (Trust & differential protection) | | Motivation patterns (Trust & differential protection) | |
|  | Trust & self-protection  OR (95% CI) | Self-protection  OR (95% CI) | Trust & self-protection  OR (95% CI) | Self-protection  OR (95% CI) | Trust & self-protection  OR (95% CI) | Self-protection  OR (95% CI) |
| Income (ref: < 5000, monthly,￥) |  |  |  |  |  |  |
| >=5000 and <10000 | 1.18* (1.04−1.35) | 1.00 (0.94−1.05) | 1.17* (1.03−1.34) | 1.06 (0.93−1.21) | 1.18* (1.03−1.35) | 1.12 (0.98−1.28) |
| >=10000 and <20000 | 1.20* (1.03−1.39) | 1.07 (0.92−1.25) | 1.17* (1.01−1.37) | 1.10 (0.95−1.28) | 1.18* (1.01−1.37) | 1.20* (1.03−1.4) |
| >=20000 and <50000 | 1.21* (1.02−1.43) | 1.12 (0.94−1.32) | 1.19* (1.00−1.41) | 1.14 (0.97−1.35) | 1.19* (1.00−1.41) | 1.23* (1.04−1.46) |
| >=50000 | 1.21* (1.03−1.43) | 1.14 (0.96−1.34) | 1.20* (1.02−1.42) | 1.19* (1.01−1.41) | 1.21* (1.02−1.43) | 1.32**(1.11−1.56) |
| Age group (ref: 25 – 34 years) |  |  |  |  |  |  |
| 35 − 44 | 1.50***(1.34−1.68) | 1.35***(1.21−1.51) | 1.46***(1.29−1.65) | 1.43***(1.27−1.62) | 1.46***(1.29−1.65) | 1.46***(1.29−1.64) |
| 45 − 54 | 1.99***(1.69−2.35) | 2.27***(1.92−2.67) | 1.93***(1.62−2.30) | 2.43***(2.04−2.89) | 1.94***(1.63−2.31) | 2.56***(2.15−3.06) |
| Gender (ref: male) |  |  |  |  |  |  |
| Female | 1.34***(1.19−1.51) | 1.12 (1.00−1.26) | 1.33***(1.18−1.51) | 1.16* (1.03−1.31) | 1.33***(1.18−1.51) | 1.15* (1.02−1.30) |
| Self-rated health | 0.96 (0.90−1.04) | 0.87***(0.81−0.93) | 0.98 (0.91−1.05) | 0.91* (0.85−0.98) | 0.98 (0.91−1.06) | 0.92* (0.86−0.99) |
| Chronic disease (Yes) | 0.97 (0.86−1.1) | 0.95 (0.84−1.07) | 0.98 (0.86−1.11) | 0.95 (0.84−1.08) | 0.97 (0.86−1.11) | 0.90 (0.79−1.02) |
| Disability (Yes) | 0.91 (0.58−1.44) | 0.85 (0.54−1.32) | 0.88 (0.56−1.39) | 0.77 (0.50−1.21) | 0.87 (0.56−1.37) | 0.76 (0.48−1.19) |
| Health behavior |  |  |  |  |  |  |
| Smoking | 0.96 (0.93−1.00) | 0.93***(0.90−0.97) | 0.97 (0.93−1.00) | 0.93**(0.90−0.97) | 0.97 (0.93−1.00) | 0.94**(0.9−0.97) |
| Drinking | 1.02 (0.97−1.08) | 0.99 (0.94−1.04) | 1.02 (0.97−1.08) | 0.99 (0.94−1.04) | 1.02 (0.97−1.08) | 0.99 (0.94−1.04) |
| Exercising | 1.06* (1.01−1.12) | 0.99 (0.94−1.05) | 1.07* (1.01−1.13) | 1.00 (0.95−1.05) | 1.07* (1.01−1.13) | 1.01 (0.96−1.06) |
| Knowledge about vaccines | 1.00 (0.94−1.05) | 0.74***(0.70−0.79) | 1.00 (0.95−1.06) | 0.75***(0.71−0.79) | 1.01 (0.95−1.06) | 0.79***(0.74−0.83) |
| Marital status (ref: single) |  |  |  |  |  |  |
| Married |  |  | 1.02 (0.89−1.18) | 0.86* (0.75−0.99) | 1.02 (0.89−1.17) | 0.84* (0.73−0.96) |
| Divorced |  |  | 0.84 (0.65−1.09) | 0.67**(0.52−0.87) | 0.85 (0.65−1.10) | 0.68**(0.52−0.88) |
| Widowed |  |  | 0.67* (0.47−0.96) | 0.60**(0.43−0.85) | 0.67* (0.47−0.95) | 0.56**(0.39−0.79) |
| Living arrangements (ref: living alone) |  |  |  |  |  |  |
| Living with family members |  |  | 1.16 (1.00−1.34) | 1.02 (0.88−1.18) | 1.16* (1.00−1.34) | 1.07 (0.92−1.23) |
| Co-rental |  |  | 1.04 (0.86−1.25) | 0.95 (0.79−1.14) | 1.04 (0.86−1.25) | 0.96 (0.80−1.15) |
| Else |  |  | 1.19 (0.96−1.46) | 0.98 (0.80−1.21) | 1.19 (0.96−1.46) | 0.97 (0.78−1.19) |
| Family relation |  |  | 0.94 (0.89−1.00) | 0.83***(0.78−0.88) | 0.95 (0.89−1.01) | 0.87***(0.82−0.92) |
| Perceptions of environmental safety |  |  |  |  |  |  |
| Government's prevention and control measures |  |  |  |  | 0.97 (0.90−1.05) | 0.89**(0.82−0.96) |
| Government's prevention and control outcomes |  |  |  |  | 0.98 (0.89−1.08) | 0.72***(0.66−0.79) |
| Concerns about the international outbreak spread |  |  |  |  | 0.99 (0.96−1.04) | 1.09***(1.04−1.13) |

Notes: **p* < 0.05; ***p* < 0.01; ****p* < 0.001

Model 1B: association between income and motivation patterns, adjusted for age, gender, self-rated health, chronic disease, disability, health behavior, and knowledge about vaccines (individual factors);

Model 2B: association between income and motivation patterns, adjusted for age, gender, self-rated health, chronic disease, disability, health behavior, and knowledge about vaccines, marital status, living arrangements, and family relation (individual and family factors);

Model 3B: association between income and motivation patterns, adjusted for age, gender, self-rated health, chronic disease, disability, health behavior, and knowledge about vaccines, marital status, living arrangements, family relation, and perceptions of environmental safety (individual, family and social factors);

S4 Table. Replication of Table 4 presenting detailed results (Model 1C-3C) on covariates.

| Variable | Model 1C | | Model 2C | | Model 3C | |
| --- | --- | --- | --- | --- | --- | --- |
|  | Motivation patterns (Trust & differential protection) | | Motivation patterns (Trust & differential protection) | | Motivation patterns (Trust & differential protection) | |
|  | Trust & self-protection  OR (95% CI) | Self-protection  OR (95% CI) | Trust & self-protection  OR (95% CI) | Self-protection  OR (95% CI) | Trust & self-protection  OR (95% CI) | Self-protection  OR (95% CI) |
| Occupation (ref: manual/unskilled service) |  |  |  |  |  |  |
| Professional/white collar | 1.01 (0.91−1.12) | 1.05 (0.95−1.16) | 1.00 (0.90−1.11) | 1.06 (0.96−1.17) | 1.00 (0.91−1.11) | 1.12* (1.02−1.25) |
| Age group (ref: 25 – 34 years) |  |  |  |  |  |  |
| 35 − 44 | 1.51***(1.35−1.69) | 1.36***(1.22−1.52) | 1.46***(1.30−1.65) | 1.43***(1.27−1.62) | 1.46***(1.30−1.65) | 1.46***(1.29−1.64) |
| 45 − 54 | 1.97***(1.67−2.33) | 2.26***(1.92−2.66) | 1.90***(1.59−2.26) | 2.41***(2.03−2.87) | 1.91***(1.60−2.27) | 2.52***(2.12−3.00) |
| Gender (ref: male) |  |  |  |  |  |  |
| Female | 1.34***(1.19−1.51) | 1.13* (1.00−1.27) | 1.33***(1.18−1.50) | 1.16* (1.03−1.31) | 1.33***(1.17−1.5) | 1.15* (1.02−1.30) |
| Self-rated health | 0.96 (0.89−1.03) | 0.87***(0.81−0.93) | 0.97 (0.90−1.05) | 0.91* (0.85−0.98) | 0.98 (0.91−1.05) | 0.92* (0.86−0.99) |
| Chronic disease (Yes) | 0.96 (0.85−1.09) | 0.95 (0.84−1.07) | 0.97 (0.85−1.10) | 0.95 (0.83−1.07) | 0.97 (0.85−1.10) | 0.89 (0.79−1.02) |
| Disability (Yes) | 0.89 (0.56−1.39) | 0.84 (0.54−1.31) | 0.86 (0.55−1.35) | 0.77 (0.49−1.20) | 0.85 (0.54−1.33) | 0.75 (0.48−1.17) |
| Health behavior |  |  |  |  |  |  |
| Smoking | 0.96 (0.93−1.00) | 0.93**(0.9−0.97) | 0.97 (0.93−1.00) | 0.94**(0.9−0.97) | 0.97 (0.93−1.00) | 0.94**(0.90−0.98) |
| Drinking | 1.03 (0.97−1.08) | 0.99 (0.94−1.04) | 1.03 (0.98−1.08) | 0.99 (0.94−1.04) | 1.03 (0.98−1.08) | 0.99 (0.94−1.05) |
| Exercising | 1.07* (1.01−1.12) | 1.00 (0.94−1.05) | 1.07* (1.01−1.13) | 1.00 (0.95−1.06) | 1.07* (1.01−1.13) | 1.01 (0.96−1.07) |
| Knowledge about vaccines | 1.00 (0.95−1.06) | 0.75***(0.71−0.79) | 1.01 (0.95−1.06) | 0.75***(0.71−0.80) | 1.01 (0.96−1.07) | 0.79***(0.75−0.83) |
| Marital status (ref: single) |  |  |  |  |  |  |
| Married |  |  | 1.03 (0.89−1.18) | 0.87* (0.76−0.99) | 1.02 (0.89−1.18) | 0.85* (0.74−0.97) |
| Divorced |  |  | 0.84 (0.64−1.08) | 0.67**(0.51−0.86) | 0.84 (0.65−1.09) | 0.68**(0.52−0.89) |
| Widowed |  |  | 0.66* (0.47−0.94) | 0.61**(0.43−0.86) | 0.66* (0.46−0.94) | 0.57**(0.40−0.80) |
| Living arrangements (ref: living alone) |  |  |  |  |  |  |
| Living with family members |  |  | 1.18* (1.02−1.37) | 1.03 (0.89−1.19) | 1.18* (1.02−1.37) | 1.08 (0.93−1.25) |
| Co-rental |  |  | 1.04 (0.86−1.26) | 0.95 (0.79−1.14) | 1.04 (0.86−1.26) | 0.96 (0.80−1.15) |
| Else |  |  | 1.17 (0.95−1.44) | 0.98 (0.80−1.20) | 1.17 (0.95−1.44) | 0.96 (0.78−1.19) |
| Family relation |  |  | 0.95 (0.89−1.01) | 0.83***(0.79−0.88) | 0.96 (0.90−1.01) | 0.87***(0.82−0.93) |
| Perceptions of environmental safety |  |  |  |  |  |  |
| Government's prevention and control measures |  |  |  |  | 0.97 (0.90−1.05) | 0.88**(0.82−0.96) |
| Government's prevention and control outcomes |  |  |  |  | 0.99 (0.90−1.08) | 0.73***(0.67−0.79) |
| Concerns about the international outbreak spread |  |  |  |  | 0.99 (0.96−1.03) | 1.09***(1.04−1.13) |

Notes: **p* < 0.05; ***p* < 0.01; ****p* < 0.001

Model 1C: association between occupation and motivation patterns, adjusted for age, gender, self-rated health, chronic disease, disability, health behavior, and knowledge about vaccines (individual factors);

Model 2C: association between occupation and motivation patterns, adjusted for age, gender, self-rated health, chronic disease, disability, health behavior, and knowledge about vaccines, marital status, living arrangements, and family relation (individual and family factors);

Model 3C: association between occupation and motivation patterns, adjusted for age, gender, self-rated health, chronic disease, disability, health behavior, and knowledge about vaccines, marital status, living arrangements, family relation, and perceptions of environmental safety (individual, family and social factors);

S5 Table. Replication of Table 4 presenting detailed results (Model 1D-3D) on covariates.

| Variable | Model 1D | | Model 2D | | Model 3D | |
| --- | --- | --- | --- | --- | --- | --- |
|  | Motivation patterns (Trust & differential protection) | | Motivation patterns (Trust & differential protection) | | Motivation patterns (Trust & differential protection) | |
|  | Trust & self-protection  OR (95% CI) | Self-protection  OR (95% CI) | Trust & self-protection  OR (95% CI) | Self-protection  OR (95% CI) | Trust & self-protection  OR (95% CI) | Self-protection  OR (95% CI) |
| Migration (ref: non-migrant) |  |  |  |  |  |  |
| Migrant | 0.96 (0.86−1.06) | 0.88* (0.79−0.98) | 0.99 (0.88−1.10) | 0.85**(0.76−0.95) | 0.98 (0.88−1.10) | 0.82**(0.74−0.92) |
| Age group (ref: 25 – 34 years) |  |  |  |  |  |  |
| 35 − 44 | 1.50***(1.34−1.68) | 1.33***(1.19−1.49) | 1.46***(1.29−1.65) | 1.41***(1.25−1.59) | 1.46***(1.29−1.65) | 1.43***(1.26−1.61) |
| 45 − 54 | 1.95***(1.64−2.31) | 2.18***(1.85−2.57) | 1.89***(1.59−2.26) | 2.33***(1.95−2.77) | 1.90***(1.59−2.27) | 2.42***(2.03−2.89) |
| Gender (ref: male) |  |  |  |  |  |  |
| Female | 1.34***(1.18−1.51) | 1.11 (0.99−1.25) | 1.33***(1.17−1.50) | 1.14* (1.01−1.29) | 1.33***(1.17−1.50) | 1.13* (1.00−1.28) |
| Self-rated health | 0.96 (0.89−1.03) | 0.87***(0.81−0.94) | 0.97 (0.90−1.05) | 0.92* (0.85−0.99) | 0.98 (0.91−1.05) | 0.93* (0.86−1.00) |
| Chronic disease (Yes) | 0.96 (0.85−1.09) | 0.95 (0.84−1.08) | 0.97 (0.86−1.10) | 0.95 (0.84−1.08) | 0.97 (0.85−1.10) | 0.90 (0.79−1.02) |
| Disability (Yes) | 0.88 (0.56−1.38) | 0.83 (0.53−1.29) | 0.86 (0.55−1.35) | 0.75 (0.48−1.18) | 0.85 (0.54−1.33) | 0.73 (0.46−1.14) |
| Health behavior |  |  |  |  |  |  |
| Smoking | 0.96 (0.93−1.00) | 0.93**(0.90−0.97) | 0.97 (0.93−1.00) | 0.94**(0.90−0.97) | 0.97 (0.93−1.00) | 0.94**(0.90−0.97) |
| Drinking | 1.03 (0.97−1.08) | 0.99 (0.94−1.04) | 1.03 (0.98−1.08) | 0.99 (0.94−1.05) | 1.03 (0.98−1.08) | 0.99 (0.94−1.05) |
| Exercising | 1.06* (1.01−1.12) | 0.99 (0.94−1.05) | 1.07* (1.01−1.13) | 1.00 (0.95−1.05) | 1.07* (1.02−1.13) | 1.01 (0.96−1.06) |
| Knowledge about vaccines | 1.00 (0.95−1.06) | 0.74***(0.71−0.79) | 1.01 (0.95−1.06) | 0.75***(0.71−0.80) | 1.01 (0.96−1.07) | 0.79***(0.75−0.83) |
| Marital status (ref: single) |  |  |  |  |  |  |
| Married |  |  | 1.03 (0.89−1.18) | 0.87* (0.76−1.00) | 1.02 (0.89−1.18) | 0.85* (0.74−0.97) |
| Divorced |  |  | 0.83 (0.64−1.08) | 0.66**(0.51−0.86) | 0.84 (0.65−1.09) | 0.67**(0.52−0.87) |
| Widowed |  |  | 0.67* (0.47−0.95) | 0.61**(0.43−0.86) | 0.66* (0.47−0.94) | 0.56**(0.40−0.80) |
| Living arrangements (ref: living alone) |  |  |  |  |  |  |
| Living with family members |  |  | 1.18* (1.02−1.37) | 0.99 (0.86−1.15) | 1.18* (1.02−1.37) | 1.04 (0.90−1.21) |
| Co-rental |  |  | 1.04 (0.86−1.26) | 0.96 (0.80−1.16) | 1.04 (0.86−1.26) | 0.98 (0.81−1.17) |
| Else |  |  | 1.17 (0.95−1.44) | 0.99 (0.80−1.21) | 1.17 (0.95−1.45) | 0.97 (0.78−1.19) |
| Family relation |  |  | 0.95 (0.89−1.01) | 0.83***(0.78−0.88) | 0.95 (0.90−1.01) | 0.87***(0.82−0.92) |
| Perceptions of environmental safety |  |  |  |  |  |  |
| Government's prevention and control measures |  |  |  |  | 0.97 (0.90−1.05) | 0.88**(0.82−0.95) |
| Government's prevention and control outcomes |  |  |  |  | 0.99 (0.90−1.08) | 0.73***(0.67−0.80) |
| Concerns about the international outbreak spread |  |  |  |  | 0.99 (0.96−1.03) | 1.09***(1.04−1.13) |

Notes: **p* < 0.05; ***p* < 0.01; ****p* < 0.001

Model 1D: association between migration and motivation patterns, adjusted for age, gender, self-rated health, chronic disease, disability, health behavior, and knowledge about vaccines (individual factors);

Model 2D: association between migration and motivation patterns, adjusted for age, gender, self-rated health, chronic disease, disability, health behavior, and knowledge about vaccines, marital status, living arrangements, and family relation (individual and family factors);

Model 3D: association between migration and motivation patterns, adjusted for age, gender, self-rated health, chronic disease, disability, health behavior, and knowledge about vaccines, marital status, living arrangements, family relation, and perceptions of environmental safety (individual, family and social factors);

S6 Table. Replication of Table 4 presenting detailed results (Model 1E-3E) on covariates.

| Variable | Model 1E | | Model 2E | | Model 3E | |
| --- | --- | --- | --- | --- | --- | --- |
|  | Motivation patterns (Trust & differential protection) | | Motivation patterns (Trust & differential protection) | | Motivation patterns (Trust & differential protection) | |
|  | Trust & self-protection  OR (95% CI) | Self-protection  OR (95% CI) | Trust & self-protection  OR (95% CI) | Self-protection  OR (95% CI) | Trust & self-protection  OR (95% CI) | Self-protection  OR (95% CI) |
| Region (ref: Chengdu) |  |  |  |  |  |  |
| Shanghai | 1.03 (0.91−1.17) | 1.32***(1.17−1.5) | 1.04 (0.91−1.17) | 1.32***(1.16−1.49) | 1.04 (0.91−1.18) | 1.35***(1.19−1.53) |
| Shenzhen | 0.79***(0.70−0.89) | 0.78***(0.69−0.88) | 0.80***(0.70−0.90) | 0.72***(0.64−0.82) | 0.79***(0.70−0.89) | 0.66***(0.58−0.75) |
| Age group (ref: 25 – 34 years) |  |  |  |  |  |  |
| 35 − 44 | 1.46***(1.31−1.63) | 1.28***(1.14−1.43) | 1.43***(1.27−1.61) | 1.36***(1.21−1.54) | 1.43***(1.27−1.61) | 1.38***(1.22−1.56) |
| 45 − 54 | 1.84***(1.55−2.18) | 2.03***(1.72−2.4) | 1.80***(1.51−2.15) | 2.18***(1.83−2.6) | 1.81***(1.51−2.16) | 2.24***(1.88−2.67) |
| Gender (ref: male) |  |  |  |  |  |  |
| Female | 1.28***(1.14−1.45) | 1.06 (0.94−1.19) | 1.28***(1.13−1.45) | 1.09 (0.97−1.24) | 1.28***(1.13−1.45) | 1.07 (0.95−1.21) |
| Self-rated health | 0.97 (0.9−1.04) | 0.87***(0.81−0.94) | 0.98 (0.91−1.06) | 0.92* (0.85−0.99) | 0.99 (0.92−1.06) | 0.94 (0.87−1.01) |
| Chronic disease (Yes) | 0.98 (0.86−1.11) | 0.95 (0.84−1.08) | 0.98 (0.87−1.11) | 0.95 (0.84−1.08) | 0.98 (0.86−1.11) | 0.89 (0.79−1.01) |
| Disability (Yes) | 0.87 (0.56−1.37) | 0.81 (0.52−1.26) | 0.84 (0.54−1.33) | 0.73 (0.47−1.14) | 0.84 (0.53−1.31) | 0.70 (0.44−1.1) |
| Health behavior |  |  |  |  |  |  |
| Smoking | 0.97 (0.93−1.01) | 0.93**(0.9−0.97) | 0.97 (0.93−1.01) | 0.94**(0.9−0.97) | 0.97 (0.93−1.01) | 0.94**(0.9−0.98) |
| Drinking | 1.02 (0.97−1.08) | 0.98 (0.93−1.03) | 1.02 (0.97−1.08) | 0.98 (0.93−1.03) | 1.02 (0.97−1.08) | 0.98 (0.93−1.03) |
| Exercising | 1.06* (1.01−1.12) | 0.99 (0.94−1.05) | 1.06* (1.01−1.12) | 1.00 (0.95−1.05) | 1.07* (1.01−1.13) | 1.01 (0.95−1.06) |
| Knowledge about vaccines | 1.00 (0.94−1.06) | 0.73***(0.7−0.78) | 1.00 (0.95−1.06) | 0.74***(0.7−0.79) | 1.01 (0.95−1.07) | 0.78***(0.74−0.83) |
| Marital status (ref: single) |  |  |  |  |  |  |
| Married |  |  | 1.02 (0.89−1.17) | 0.85* (0.75−0.98) | 1.02 (0.89−1.17) | 0.83**(0.72−0.95) |
| Divorced |  |  | 0.82 (0.63−1.06) | 0.64**(0.49−0.83) | 0.83 (0.64−1.07) | 0.64**(0.49−0.84) |
| Widowed |  |  | 0.69* (0.49−0.98) | 0.63**(0.45−0.89) | 0.68* (0.48−0.97) | 0.58**(0.41−0.83) |
| Living arrangements (ref: living alone) |  |  |  |  |  |  |
| Living with family members |  |  | 1.11 (0.96−1.29) | 0.94 (0.81−1.09) | 1.12 (0.96−1.3) | 0.97 (0.84−1.13) |
| Co-rental |  |  | 0.99 (0.82−1.19) | 0.84 (0.7−1.02) | 0.98 (0.81−1.19) | 0.83 (0.69−1) |
| Else |  |  | 1.17 (0.95−1.45) | 0.97 (0.79−1.2) | 1.17 (0.95−1.44) | 0.95 (0.77−1.17) |
| Family relation |  |  | 0.94* (0.88−1) | 0.82***(0.77−0.87) | 0.94 (0.89−1) | 0.86***(0.81−0.91) |
| Perceptions of environmental safety |  |  |  |  |  |  |
| Government's prevention and control measures |  |  |  |  | 0.97 (0.89−1.05) | 0.88**(0.81−0.95) |
| Government's prevention and control outcomes |  |  |  |  | 0.97 (0.89−1.07) | 0.70***(0.64−0.77) |
| Concerns about the international outbreak spread |  |  |  |  | 1.00 (0.96−1.04) | 1.09***(1.05−1.14) |

Notes: **p* < 0.05; ***p* < 0.01; ****p* < 0.001

Model 1E: association between region and motivation patterns, adjusted for age, gender, self-rated health, chronic disease, disability, health behavior, and knowledge about vaccines (individual factors);

Model 2E: association between region and motivation patterns, adjusted for age, gender, self-rated health, chronic disease, disability, health behavior, and knowledge about vaccines, marital status, living arrangements, and family relation (individual and family factors);

Model 3E: association between region and motivation patterns, adjusted for age, gender, self-rated health, chronic disease, disability, health behavior, and knowledge about vaccines, marital status, living arrangements, family relation, and perceptions of environmental safety (individual, family and social factors);

S7 Table. A multivariable multinomial logistic regression model with the categorical dependent variable (vaccination motivation): effects after mutual adjustment.

| Variable | Model 1 | | Model 2 | | Model 3 | |
| --- | --- | --- | --- | --- | --- | --- |
|  | Motivation patterns (Trust & differential protection) | | Motivation patterns (Trust & differential protection) | | Motivation patterns (Trust & differential protection) | |
|  | Trust & self-protection  OR (95% CI) | Self-protection  OR (95% CI) | Trust & self-protection  OR (95% CI) | Self-protection  OR (95% CI) | Trust & self-protection  OR (95% CI) | Self-protection  OR (95% CI) |
| Education (ref: illiteracy and primary school) |  |  |  |  |  |  |
| Junior high school | 1.94**(1.23−3.06) | 0.68 (0.46−1.00) | 1.96** (1.24−3.08) | 0.71 (0.48−1.04) | 1.95**(1.23−3.07) | 0.72 (0.49−1.07) |
| Senior high school | 1.84**(1.17−2.91) | 0.55**(0.37−0.82) | 1.87**(1.18−2.95) | 0.58**(0.39−0.85) | 1.86**(1.18−2.94) | 0.61*(0.41−0.91) |
| University and junior college | 1.79* (1.13−2.84) | 0.59* (0.40−0.88) | 1.83* (1.15−2.90) | 0.62* (0.42−0.93) | 1.82* (1.15−2.89) | 0.68 (0.45−1.01) |
| Master and above | 1.94**(1.18−3.18) | 0.79 (0.51−1.21) | 1.99**(1.21−3.27) | 0.80 (0.52−1.24) | 1.98**(1.20−3.26) | 0.89 (0.57−1.39) |
| Income (ref: < 5000, monthly,￥) |  |  |  |  |  |  |
| >=5000 and <10000 | 1.16* (1.01−1.33) | 1.01 (0.88−1.15) | 1.15* (1.01−1.32) | 1.03 (0.90−1.18) | 1.16* (1.01−1.33) | 1.06 (0.93−1.22) |
| >=10000 and <20000 | 1.16 (0.99−1.37) | 0.99 (0.84−1.16) | 1.15 (0.97−1.35) | 1.02 (0.87−1.20) | 1.15 (0.98−1.35) | 1.07 (0.91−1.26) |
| >=20000 and <50000 | 1.17 (0.98−1.40) | 1.02 (0.86−1.22) | 1.16 (0.97−1.38) | 1.05 (0.88−1.25) | 1.16 (0.97−1.39) | 1.09 (0.91−1.30) |
| >=50000 | 1.17 (0.98−1.40) | 1.04 (0.87−1.23) | 1.17 (0.98−1.39) | 1.08 (0.91−1.29) | 1.17 (0.98−1.40) | 1.15 (0.96−1.37) |
| Occupation (ref: manual/unskilled service) |  |  |  |  |  |  |
| professional/white collar | 0.92 (0.83−1.03) | 0.95 (0.85−1.07) | 0.92 (0.82−1.03) | 0.94 (0.84−1.06) | 0.92 (0.83−1.03) | 0.96 (0.86−1.08) |
| Migration (ref: non-migrant) |  |  |  |  |  |  |
| Migrant | 1.06 (0.94−1.20) | 0.91 (0.80−1.03) | 1.08 (0.95−1.22) | 0.89 (0.78−1.01) | 1.08 (0.95−1.22) | 0.89 (0.79−1.01) |
| Region (ref: Chengdu) |  |  |  |  |  |  |
| Shanghai | 0.99 (0.87−1.13) | 1.35***(1.18−1.54) | 0.99 (0.87−1.14) | 1.35***(1.18−1.54) | 1.00 (0.87−1.14) | 1.39***(1.21−1.59) |
| Shenzhen | 0.76***(0.66−0.87) | 0.82**(0.71−0.95) | 0.76***(0.66−0.88) | 0.78**(0.67−0.90) | 0.75***(0.65−0.87) | 0.73***(0.63−0.84) |
| Age group (ref: 25 – 34 years) |  |  |  |  |  |  |
| 35 − 44 | 1.48***(1.32−1.66) | 1.25***(1.12−1.41) | 1.45***(1.29−1.64) | 1.33***(1.18−1.51) | 1.45***(1.29−1.64) | 1.35***(1.19−1.53) |
| 45 − 54 | 1.91***(1.60−2.29) | 1.91***(1.61−2.28) | 1.89***(1.57−2.27) | 2.05***(1.71−2.46) | 1.90***(1.58−2.28) | 2.17***(1.80−2.61) |
| Gender (ref: male) |  |  |  |  |  |  |
| Female | 1.29***(1.14−1.46) | 1.04 (0.92−1.17) | 1.30***(1.14−1.47) | 1.07* (0.95−1.21) | 1.29***(1.14−1.46) | 1.06 (0.94−1.20) |
| Self-rated health | 0.96 (0.90−1.04) | 0.87***(0.81−0.94) | 0.98 (0.91−1.06) | 0.92* (0.85−0.99) | 0.99 (0.91−1.06) | 0.94 (0.87−1.01) |
| Chronic disease (Yes) | 0.99 (0.87−1.12) | 0.95 (0.84−1.08) | 0.99 (0.87−1.12) | 0.95 (0.84−1.08) | 0.98 (0.87−1.12) | 0.90 (0.79−1.03) |
| Disability (Yes) | 0.90 (0.57−1.42) | 0.77 (0.49−1.21) | 0.87 (0.55−1.37) | 0.70 (0.45−1.10) | 0.86 (0.54−1.35) | 0.69 (0.44−1.09) |
| Health behavior |  |  |  |  |  |  |
| Smoking | 0.96 (0.93−1.00) | 0.94**(0.90−0.98) | 0.97 (0.93−1.01) | 0.94**(0.90−0.98) | 0.97 (0.93−1.01) | 0.95**(0.91−0.98) |
| Drinking | 1.02 (0.96−1.07) | 0.98 (0.93−1.04) | 1.02 (0.97−1.08) | 0.98 (0.93−1.04) | 1.02 (0.96−1.07) | 0.98 (0.93−1.03) |
| Exercising | 1.06* (1.00−1.12) | 0.99 (0.94−1.05) | 1.06* (1.01−1.12) | 1.00 (0.95−1.05) | 1.06* (1.01−1.12) | 1.00 (0.95−1.06) |
| Knowledge about vaccines | 1.00 (0.94−1.06) | 0.74***(0.70−0.78) | 1.00 (0.95−1.06) | 0.75***(0.71−0.79) | 1.01 (0.95−1.07) | 0.78***(0.74−0.83) |
| Marital status (ref: single) |  |  |  |  |  |  |
| Married |  |  | 1.01 (0.88−1.16) | 0.86* (0.75−0.99) | 1.01 (0.88−1.16) | 0.85* (0.74−0.97) |
| Divorced |  |  | 0.82 (0.63−1.06) | 0.64**(0.49−0.83) | 0.83 (0.64−1.07) | 0.66**(0.51−0.86) |
| Widowed |  |  | 0.68* (0.48−0.97) | 0.63**(0.44−0.89) | 0.67* (0.47−0.96) | 0.60**(0.42−0.85) |
| Living arrangements (ref: living alone) |  |  |  |  |  |  |
| Living with family members |  |  | 1.12 (0.96−1.30) | 0.93 (0.80−1.07) | 1.12 (0.96−1.30) | 0.95 (0.82−1.11) |
| Co-rental |  |  | 0.98 (0.81−1.19) | 0.86 (0.71−1.03) | 0.98 (0.81−1.18) | 0.85 (0.70−1.03) |
| Else |  |  | 1.16 (0.94−1.44) | 0.96 (0.78−1.19) | 1.16 (0.94−1.44) | 0.95 (0.77−1.18) |
| Family relation |  |  | 0.93* (0.88−0.99) | 0.82***(0.77−0.87) | 0.94 (0.88−1.00) | 0.86***(0.81−0.91) |
| Perceptions of environmental safety |  |  |  |  |  |  |
| Government's prevention and control measures |  |  |  |  | 0.97 (0.89−1.05) | 0.88**(0.81−0.95) |
| Government's prevention and control outcomes |  |  |  |  | 0.97 (0.88−1.06) | 0.71***(0.65−0.77) |
| Concerns about the international outbreak spread |  |  |  |  | 1.00 (0.96−1.04) | 1.09***(1.05−1.14) |

Notes: **p* < 0.05; ***p* < 0.01; ****p* < 0.001

S8 Table. Motivation patterns multinominal logistic regression: gender as an intersectional variable (n = 12,432).

| Variable | Model 1F-1J | | Model 2F-2J | | Model 3F-3J | |
| --- | --- | --- | --- | --- | --- | --- |
|  | Motivation patterns (Trust & differential protection) | | Motivation patterns (Trust & differential protection) | | Motivation patterns (Trust & differential protection) | |
|  | Trust & self-protection  OR (95% CI) | Self-protection  OR (95% CI) | Trust & self-protection  OR (95% CI) | Self-protection  OR (95% CI) | Trust & self-protection  OR (95% CI) | Self-protection  OR (95% CI) |
| ***Panel F: Independent variable: education, gender as an intersectional variable*** | | | | | | |
| Education (ref: illiteracy and primary school) |  |  |  |  |  |  |
| Junior high school | 3.01**(1.43−6.37) | 0.73 (0.42−1.27) | 3.02**(1.43−6.39) | 0.74 (0.43−1.29) | 3.01**(1.43−6.37) | 0.75 (0.43−1.32) |
| Senior high school | 2.74**(1.30−5.77) | 0.61 (0.36−1.06) | 2.77**(1.31−5.83) | 0.63 (0.37−1.09) | 2.76**(1.31−5.82) | 0.67 (0.39−1.17) |
| University and junior college | 3.06**(1.45−6.44) | 0.67 (0.39−1.16) | 3.04**(1.44−6.41) | 0.72 (0.41−1.23) | 3.05**(1.45−6.42) | 0.80 (0.46−1.39) |
| Master and above | 4.00***(1.84−8.70) | 1.04 (0.58−1.87) | 4.01***(1.84−8.73) | 1.11 (0.62−2.00) | 4.02***(1.85−8.75) | 1.30 (0.72−2.36) |
| Gender (ref: male) |  |  |  |  |  |  |
| Female | 2.80*(1.10−7.17) | 1.37 (0.65−2.90) | 2.78*(1.09−7.11) | 1.39 (0.66−2.95) | 2.78*(1.09−7.11) | 1.38 (0.65−2.94) |
| Education * Gender |  |  |  |  |  |  |
| Junior high school * Female | 0.47 (0.18−1.24) | 0.79 (0.37−1.71) | 0.48 (0.18−1.24) | 0.82 (0.38−1.78) | 0.48 (0.18−1.24) | 0.83 (0.38−1.82) |
| Senior high school* Female | 0.55 (0.21−1.43) | 0.70 (0.32−1.52) | 0.54 (0.21−1.41) | 0.72 (0.33−1.55) | 0.54 (0.21−1.41) | 0.71 (0.32−1.55) |
| University and junior college * Female | 0.48 (0.19−1.24) | 0.90 (0.42−1.92) | 0.48 (0.19−1.25) | 0.90 (0.42−1.92) | 0.48 (0.19−1.25) | 0.92 (0.42−1.98) |
| Master and above * Female | 0.33*(0.12−0.91) | 0.72 (0.32−1.65) | 0.34*(0.12−0.92) | 0.71 (0.31−1.62) | 0.34*(0.12−0.92) | 0.71 (0.31−1.64) |
| ***Panel G: Independent variable: income, gender as an intersectional variable*** | | | | | | |
| Income (ref: < 5000, monthly,￥) |  |  |  |  |  |  |
| >=5000 and <10000 | 1.21*(1.02−1.43) | 0.93 (0.79−1.10) | 1.19*(1.00−1.41) | 0.96 (0.82−1.14) | 1.20*(1.01−1.42) | 1.03 (0.87−1.22) |
| >=10000 and <20000 | 1.35**(1.10−1.65) | 1.06 (0.87−1.30) | 1.32**(1.07−1.62) | 1.11 (0.91−1.35) | 1.32**(1.08−1.62) | 1.22 (0.99−1.49) |
| >=20000 and <50000 | 1.39**(1.11−1.75) | 1.07 (0.86−1.34) | 1.37**(1.09−1.72) | 1.10 (0.88−1.38) | 1.37**(1.09−1.72) | 1.19 (0.94−1.49) |
| >=50000 | 1.19 (0.96−1.48) | 0.94 (0.76−1.16) | 1.18 (0.95−1.47) | 1.00 (0.80−1.23) | 1.18 (0.95−1.47) | 1.10 (0.89−1.36) |
| Gender (ref: male) |  |  |  |  |  |  |
| Female | 1.46***(1.19−1.78) | 0.97 (0.80−1.18) | 1.44**(1.17−1.77) | 1.02 (0.83−1.24) | 1.44**(1.17−1.76) | 1.02 (0.83−1.25) |
| Income * Gender |  |  |  |  |  |  |
| >=5000 and <10000 * Female | 1.00 (0.76−1.30) | 1.30 (1.00−1.70) | 1.00 (0.77−1.31) | 1.27 (0.97−1.66) | 1.00 (0.77−1.31) | 1.24 (0.95−1.63) |
| >=10000 and <20000 * Female | 0.77 (0.56−1.04) | 1.01 (0.75−1.36) | 0.77 (0.57−1.05) | 0.98 (0.72−1.32) | 0.78 (0.57−1.06) | 0.97 (0.71−1.31) |
| >=20000 and <50000 * Female | 0.74 (0.53−1.04) | 1.09 (0.78−1.52) | 0.75 (0.54−1.05) | 1.08 (0.78−1.52) | 0.75 (0.54−1.05) | 1.09 (0.77−1.52) |
| >=50000 * Female | 1.09 (0.78−1.53) | 1.59**(1.14−2.22) | 1.10 (0.78−1.54) | 1.54*(1.10−2.15) | 1.10 (0.79−1.54) | 1.54*(1.10−2.16) |
| ***Panel H: Independent variable: occupation, gender as an intersectional variable*** | | | | | | |
| Occupation (ref: manual/unskilled service) |  |  |  |  |  |  |
| professional/white collar | 1.03 (0.90−1.18) | 0.98 (0.87−1.12) | 1.02 (0.89−1.16) | 1.00 (0.88−1.13) | 1.02 (0.89−1.16) | 1.06 (0.93−1.21) |
| Gender (ref: male) |  |  |  |  |  |  |
| Female | 1.37***(1.16−1.62) | 1.03 (0.87−1.21) | 1.35**(1.14−1.61) | 1.06 (0.90−1.26) | 1.35**(1.14−1.60) | 1.06 (0.89−1.26) |
| Occupation * Gender |  |  |  |  |  |  |
| professional/white collar * Female | 0.97 (0.79−1.19) | 1.18 (0.96−1.44) | 0.97 (0.79−1.19) | 1.16 (0.95−1.42) | 0.97 (0.79−1.19) | 1.15 (0.94−1.41) |
| ***Panel I: Independent variable: migration, gender as an intersectional variable*** | | | | | | |
| Migration (ref: non-migrant) |  |  |  |  |  |  |
| Migrant | 0.91 (0.79−1.05) | 0.82**(0.72−0.95) | 0.94 (0.81−1.09) | 0.79**(0.68−0.91) | 0.94 (0.81−1.09) | 0.75***(0.65−0.87) |
| Gender (ref: male) |  |  |  |  |  |  |
| Female | 1.24*(1.05−1.47) | 1.02 (0.86−1.2) | 1.24*(1.05−1.48) | 1.03 (0.87−1.22) | 1.24*(1.04−1.47) | 1.01 (0.85−1.2) |
| Migration * Gender |  |  |  |  |  |  |
| Migrant* Female | 1.13 (0.92−1.39) | 1.16 (0.95−1.43) | 1.12 (0.91−1.38) | 1.20 (0.97−1.47) | 1.12 (0.91−1.38) | 1.22 (0.99−1.5) |
| ***Panel J: Independent variable: region, gender as an intersectional variable*** | | | | | | |
| Region (ref: Chengdu) |  |  |  |  |  |  |
| Shanghai | 1.02 (0.86−1.21) | 1.10 (0.93−1.30) | 1.02 (0.86−1.21) | 1.10 (0.93−1.30) | 1.02 (0.86−1.21) | 1.12 (0.94−1.32) |
| Shenzhen | 0.77**(0.66−0.90) | 0.79**(0.67−0.92) | 0.77**(0.66−0.91) | 0.72***(0.61−0.84) | 0.77**(0.65−0.90) | 0.65***(0.56−0.77) |
| Gender (ref: male) |  |  |  |  |  |  |
| Female | 1.25*(1.05−1.50) | 0.96 (0.80−1.14) | 1.25*(1.05−1.49) | 0.97 (0.81−1.15) | 1.24*(1.04−1.48) | 0.94 (0.78−1.12) |
| Region * Gender |  |  |  |  |  |  |
| Shanghai* Female | 1.04 (0.81−1.34) | 1.47**(1.15−1.89) | 1.05 (0.82−1.35) | 1.47**(1.15−1.88) | 1.05 (0.82−1.35) | 1.50**(1.17−1.93) |
| Shenzhen* Female | 1.07 (0.84−1.37) | 0.90 (0.70−1.15) | 1.07 (0.84−1.37) | 0.96 (0.75−1.24) | 1.07 (0.84−1.37) | 0.96 (0.75−1.24) |

Notes: **p* < 0.05; ***p* < 0.01; ****p* < 0.001

Model 1F-1J: association between education (income/occupation/migration/region) and motivation patterns, gender as an intersectional variable, adjusted for age, self-rated health, health behavior, and knowledge about vaccines (individual factors);

Model 2F-2J: association between education (income/occupation/migration/region) and motivation patterns, gender as an intersectional variable, adjusted for age, self-rated health, health behavior, and knowledge about vaccines, marital status, living arrangements, and family relation (individual and family factors);

Model 3F-3J: association between education (income/occupation/migration/region) and motivation patterns, gender as an intersectional variable, adjusted for age, self-rated health, health behavior, and knowledge about vaccines, marital status, living arrangements, family relation, and perceptions of environmental safety (individual, family and social factors)

S9 Table. Replication of S8 presenting detailed results (Model 1F-3F) on covariates.

| Variable | Model 1F | | Model 2F | | Model 3F | |
| --- | --- | --- | --- | --- | --- | --- |
|  | Motivation patterns (Trust & differential protection) | | Motivation patterns (Trust & differential protection) | | Motivation patterns (Trust & differential protection) | |
|  | Trust & self-protection  OR (95% CI) | Self-protection  OR (95% CI) | Trust & self-protection  OR (95% CI) | Self-protection  OR (95% CI) | Trust & self-protection  OR (95% CI) | Self-protection  OR (95% CI) |
| Education (ref: illiteracy and primary school) |  |  |  |  |  |  |
| Junior high school | 3.01**(1.43−6.37) | 0.73 (0.42−1.27) | 3.02**(1.43−6.39) | 0.74 (0.43−1.29) | 3.01**(1.43−6.37) | 0.75 (0.43−1.32) |
| Senior high school | 2.74**(1.30−5.77) | 0.61 (0.36−1.06) | 2.77**(1.31−5.83) | 0.63 (0.37−1.09) | 2.76**(1.31−5.82) | 0.67 (0.39−1.17) |
| University and junior college | 3.06**(1.45−6.44) | 0.67 (0.39−1.16) | 3.04**(1.44−6.41) | 0.72 (0.41−1.23) | 3.05**(1.45−6.42) | 0.80 (0.46−1.39) |
| Master and above | 4.00***(1.84−8.70) | 1.04 (0.58−1.87) | 4.01***(1.84−8.73) | 1.11 (0.62−2.00) | 4.02***(1.85−8.75) | 1.30 (0.72−2.36) |
| Gender (ref: male) |  |  |  |  |  |  |
| Female | 2.80* (1.10−7.17) | 1.37 (0.65−2.90) | 2.78* (1.09−7.11) | 1.39 (0.66−2.95) | 2.78* (1.09−7.11) | 1.38 (0.65−2.94) |
| Education * Gender |  |  |  |  |  |  |
| Junior high school * Female | 0.47 (0.18−1.24) | 0.79 (0.37−1.71) | 0.48 (0.18−1.24) | 0.82 (0.38−1.78) | 0.48 (0.18−1.24) | 0.83 (0.38−1.82) |
| Senior high school* Female | 0.55 (0.21−1.43) | 0.70 (0.32−1.52) | 0.54 (0.21−1.41) | 0.72 (0.33−1.55) | 0.54 (0.21−1.41) | 0.71 (0.32−1.55) |
| University and junior college * Female | 0.48 (0.19−1.24) | 0.90 (0.42−1.92) | 0.48 (0.19−1.25) | 0.90 (0.42−1.92) | 0.48 (0.19−1.25) | 0.92 (0.42−1.98) |
| Master and above * Female | 0.33* (0.12−0.91) | 0.72 (0.32−1.65) | 0.34* (0.12−0.92) | 0.71 (0.31−1.62) | 0.34* (0.12−0.92) | 0.71 (0.31−1.64) |
| Age group (ref: 25 – 34 years) |  |  |  |  |  |  |
| 35 − 44 | 1.51***(1.35−1.69) | 1.36***(1.22−1.52) | 1.47***(1.30−1.66) | 1.42***(1.26−1.60) | 1.47***(1.30−1.66) | 1.45***(1.28−1.64) |
| 45 − 54 | 2.03***(1.71−2.41) | 2.21***(1.87−2.62) | 1.96***(1.64−2.34) | 2.35***(1.97−2.80) | 1.97***(1.65−2.35) | 2.51***(2.10−3.00) |
| Self-rated health | 0.97 (0.90−1.04) | 0.87***(0.81−0.93) | 0.98 (0.91−1.06) | 0.91* (0.84−0.98) | 0.98 (0.91−1.06) | 0.93* (0.86−1.00) |
| Chronic disease (Yes) | 0.97 (0.86−1.10) | 0.96 (0.84−1.08) | 0.98 (0.86−1.11) | 0.96 (0.84−1.08) | 0.98 (0.86−1.11) | 0.91 (0.80−1.03) |
| Disability (Yes) | 0.94 (0.60−1.48) | 0.82 (0.53−1.28) | 0.91 (0.58−1.43) | 0.75 (0.48−1.18) | 0.89 (0.56−1.40) | 0.74 (0.47−1.16) |
| Health behavior |  |  |  |  |  |  |
| Smoking | 0.97 (0.94−1.01) | 0.94**(0.91−0.98) | 0.97 (0.94−1.02) | 0.95**(0.91−0.99) | 0.98 (0.94−1.02) | 0.95* (0.92−0.99) |
| Drinking | 1.02 (0.97−1.08) | 0.99 (0.94−1.04) | 1.03 (0.97−1.08) | 0.99 (0.94−1.04) | 1.02 (0.97−1.08) | 0.99 (0.94−1.04) |
| Exercising | 1.06* (1.00−1.12) | 0.99 (0.94−1.05) | 1.06* (1.01−1.12) | 1.00 (0.95−1.05) | 1.06* (1.01−1.12) | 1.00 (0.95−1.06) |
| Knowledge about vaccines | 1.00 (0.95−1.06) | 0.75***(0.71−0.79) | 1.00 (0.95−1.06) | 0.76***(0.72−0.80) | 1.01 (0.95−1.07) | 0.79***(0.75−0.84) |
| Marital status (ref: single) |  |  |  |  |  |  |
| Married |  |  | 1.03 (0.9−1.19) | 0.90 (0.78−1.03) | 1.03 (0.90−1.19) | 0.88 (0.77−1.02) |
| Divorced |  |  | 0.84 (0.65−1.09) | 0.69**(0.53−0.90) | 0.85 (0.65−1.10) | 0.71* (0.55−0.93) |
| Widowed |  |  | 0.68* (0.47−0.96) | 0.63**(0.44−0.89) | 0.67* (0.47−0.95) | 0.6** (0.42−0.85) |
| Living arrangements (ref: living alone) |  |  |  |  |  |  |
| Living with family members |  |  | 1.16* (1.00−1.34) | 1.02 (0.88−1.17) | 1.16* (1.01−1.35) | 1.06 (0.92−1.23) |
| Co-rental |  |  | 1.04 (0.86−1.26) | 0.95 (0.79−1.14) | 1.04 (0.86−1.26) | 0.96 (0.8−1.15) |
| Else |  |  | 1.17 (0.95−1.45) | 0.97 (0.79−1.20) | 1.17 (0.95−1.45) | 0.96 (0.78−1.19) |
| Family relation |  |  | 0.94 (0.89−1.00) | 0.83***(0.78−0.88) | 0.95 (0.89−1.01) | 0.87***(0.82−0.92) |
| Perceptions of environmental safety |  |  |  |  |  |  |
| Government's prevention and control measures |  |  |  |  | 0.97 (0.89−1.05) | 0.88 (0.81−0.95) |
| Government's prevention and control outcomes |  |  |  |  | 0.98 (0.9−1.08) | 0.72***(0.66−0.79) |
| Concerns about the international outbreak spread |  |  |  |  | 0.99 (0.96−1.04) | 1.09***(1.05−1.14) |

Notes: **p* < 0.05; ***p* < 0.01; ****p* < 0.001

Model 1F: association between education and motivation patterns, gender as an intersectional variable, adjusted for age, self-rated health, chronic disease, disability, health behavior, and knowledge about vaccines (individual factors);

Model 2F: association between education and motivation patterns, gender as an intersectional variable, adjusted for age, self-rated health, chronic disease, disability, health behavior, and knowledge about vaccines, marital status, living arrangements, and family relation (individual and family factors);

Model 3F: association between education and motivation patterns, gender as an intersectional variable, adjusted for age, self-rated health, chronic disease, disability, health behavior, and knowledge about vaccines, marital status, living arrangements, family relation, and perceptions of environmental safety (individual, family and social factors);

S10 Table. Replication of S8 presenting detailed results (Model 1G-3G) on covariates.

| Variable | Model 1G | | Model 2G | | Model 3G | |
| --- | --- | --- | --- | --- | --- | --- |
|  | Motivation patterns (Trust & differential protection) | | Motivation patterns (Trust & differential protection) | | Motivation patterns (Trust & differential protection) | |
|  | Trust & self-protection  OR (95% CI) | Self-protection  OR (95% CI) | Trust & self-protection  OR (95% CI) | Self-protection  OR (95% CI) | Trust & self-protection  OR (95% CI) | Self-protection  OR (95% CI) |
| Income (ref: < 5000, monthly,￥) |  |  |  |  |  |  |
| >=5000 and <10000 | 1.21* (1.02−1.43) | 0.93 (0.79−1.10) | 1.19* (1.00−1.41) | 0.96 (0.82−1.14) | 1.20* (1.01−1.42) | 1.03 (0.87−1.22) |
| >=10000 and <20000 | 1.35**(1.10−1.65) | 1.06 (0.87−1.30) | 1.32**(1.07−1.62) | 1.11 (0.91−1.35) | 1.32**(1.08−1.62) | 1.22 (0.99−1.49) |
| >=2000 and <50000 | 1.39**(1.11−1.75) | 1.07 (0.86−1.34) | 1.37**(1.09−1.72) | 1.10 (0.88−1.38) | 1.37**(1.09−1.72) | 1.19 (0.94−1.49) |
| >=50000 | 1.19 (0.96−1.48) | 0.94 (0.76−1.16) | 1.18 (0.95−1.47) | 1.00 (0.80−1.23) | 1.18 (0.95−1.47) | 1.10 (0.89−1.36) |
| Gender (ref: male) |  |  |  |  |  |  |
| Female | 1.46***(1.19−1.78) | 0.97 (0.80−1.18) | 1.44**(1.17−1.77) | 1.02 (0.83−1.24) | 1.44**(1.17−1.76) | 1.02 (0.83−1.25) |
| Income * Gender |  |  |  |  |  |  |
| >=5000 and <10000 * Female | 1.00 (0.76−1.30) | 1.30 (1.00−1.70) | 1.00 (0.77−1.31) | 1.27 (0.97−1.66) | 1.00 (0.77−1.31) | 1.24 (0.95−1.63) |
| >=10000 and <20000 * Female | 0.77 (0.56−1.04) | 1.01 (0.75−1.36) | 0.77 (0.57−1.05) | 0.98 (0.72−1.32) | 0.78 (0.57−1.06) | 0.97 (0.71−1.31) |
| >=20000 and <50000 * Female | 0.74 (0.53−1.04) | 1.09 (0.78−1.52) | 0.75 (0.54−1.05) | 1.08 (0.78−1.52) | 0.75 (0.54−1.05) | 1.09 (0.77−1.52) |
| >=50000 * Female | 1.09 (0.78−1.53) | 1.59**(1.14−2.22) | 1.10 (0.78−1.54) | 1.54* (1.10−2.15) | 1.10 (0.79−1.54) | 1.54* (1.10−2.16) |
| Age group (ref: 25 – 34 years) |  |  |  |  |  |  |
| 35 − 44 | 1.50***(1.34−1.67) | 1.35***(1.21−1.51) | 1.46***(1.29−1.65) | 1.43***(1.27−1.61) | 1.46***(1.29−1.65) | 1.45***(1.28−1.64) |
| 45 − 54 | 1.99***(1.69−2.35) | 2.28***(1.93−2.68) | 1.93***(1.62−2.30) | 2.43***(2.04−2.89) | 1.94***(1.63−2.32) | 2.57***(2.15−3.06) |
| Self-rated health | 0.96 (0.90−1.04) | 0.87***(0.81−0.93) | 0.98 (0.91−1.05) | 0.91* (0.85−0.98) | 0.98 (0.91−1.06) | 0.92* (0.86−0.99) |
| Chronic disease (Yes) | 0.97 (0.86−1.10) | 0.95 (0.84−1.07) | 0.98 (0.86−1.11) | 0.95 (0.84−1.07) | 0.97 (0.86−1.11) | 0.90 (0.79−1.02) |
| Disability (Yes) | 0.93 (0.59−1.46) | 0.85 (0.54−1.32) | 0.90 (0.57−1.41) | 0.77 (0.50−1.21) | 0.89 (0.56−1.39) | 0.76 (0.48−1.19) |
| Health behavior |  |  |  |  |  |  |
| Smoking | 0.97 (0.93−1.01) | 0.93***(0.90−0.97) | 0.97 (0.93−1.01) | 0.94**(0.9−0.97) | 0.97 (0.93−1.01) | 0.94**(0.90−0.97) |
| Drinking | 1.02 (0.97−1.08) | 0.99 (0.94−1.04) | 1.02 (0.97−1.08) | 0.99 (0.94−1.04) | 1.02 (0.97−1.08) | 0.99 (0.94−1.04) |
| Exercising | 1.06* (1.01−1.12) | 1.00 (0.94−1.05) | 1.07* (1.01−1.12) | 1.00 (0.95−1.06) | 1.07* (1.01−1.13) | 1.01 (0.96−1.07) |
| Knowledge about vaccines | 1.00 (0.94−1.06) | 0.74***(0.70−0.79) | 1.00 (0.95−1.06) | 0.75***(0.71−0.79) | 1.01 (0.95−1.07) | 0.79***(0.74−0.83) |
| Marital status (ref: single) |  |  |  |  |  |  |
| Married |  |  | 1.02 (0.89−1.17) | 0.87* (0.76−1.00) | 1.02 (0.89−1.17) | 0.85* (0.74−0.97) |
| Divorced |  |  | 0.84 (0.65−1.10) | 0.68**(0.52−0.88) | 0.85 (0.66−1.10) | 0.69**(0.53−0.90) |
| Widowed |  |  | 0.67* (0.47−0.95) | 0.61**(0.43−0.86) | 0.66* (0.47−0.94) | 0.56**(0.40−0.80) |
| Living arrangements (ref: living alone) |  |  |  |  |  |  |
| Living with family members |  |  | 1.16 (1.00−1.34) | 1.02 (0.88−1.18) | 1.16* (1.00−1.34) | 1.07 (0.92−1.23) |
| Co-rental |  |  | 1.03 (0.86−1.25) | 0.96 (0.80−1.15) | 1.03 (0.85−1.25) | 0.96 (0.80−1.16) |
| Else |  |  | 1.19 (0.96−1.47) | 0.99 (0.80−1.21) | 1.19 (0.96−1.47) | 0.97 (0.79−1.20) |
| Family relation |  |  | 0.94 (0.89−1.00) | 0.83***(0.78−0.88) | 0.95 (0.89−1.01) | 0.87***(0.82−0.92) |
| Perceptions of environmental safety |  |  |  |  |  |  |
| Government's prevention and control measures |  |  |  |  | 0.97 (0.90−1.05) | 0.89**(0.82−0.96) |
| Government's prevention and control outcomes |  |  |  |  | 0.98 (0.89−1.07) | 0.72***(0.66−0.79) |
| Concerns about the international outbreak spread |  |  |  |  | 0.99 (0.96−1.04) | 1.09***(1.05−1.13) |

Notes: **p* < 0.05; ***p* < 0.01; ****p* < 0.001

Model 1G: association between income and motivation patterns, gender as an intersectional variable, adjusted for age, self-rated health, chronic disease, disability, health behavior, and knowledge about vaccines (individual factors);

Model 2G: association between income and motivation patterns, gender as an intersectional variable, adjusted for age, self-rated health, chronic disease, disability, health behavior, and knowledge about vaccines, marital status, living arrangements, and family relation (individual and family factors);

Model 3G: association between income and motivation patterns, gender as an intersectional variable, adjusted for age, self-rated health, chronic disease, disability, health behavior, and knowledge about vaccines, marital status, living arrangements, family relation, and perceptions of environmental safety (individual, family and social factors);

S11 Table. Replication of S8 presenting detailed results (Model 1H-3H) on covariates.

| Variable | Model 1H | | Model 2H | | Model 3H | |
| --- | --- | --- | --- | --- | --- | --- |
|  | Motivation patterns (Trust & differential protection) | | Motivation patterns (Trust & differential protection) | | Motivation patterns (Trust & differential protection) | |
|  | Trust & self-protection  OR (95% CI) | Self-protection  OR (95% CI) | Trust & self-protection  OR (95% CI) | Self-protection  OR (95% CI) | Trust & self-protection  OR (95% CI) | Self-protection  OR (95% CI) |
| Occupation (ref: manual/unskilled service) |  |  |  |  |  |  |
| professional/white collar | 1.03 (0.90−1.18) | 0.98 (0.87−1.12) | 1.02 (0.89−1.16) | 1.00 (0.88−1.13) | 1.02 (0.89−1.16) | 1.06 (0.93−1.21) |
| Gender (ref: male) |  |  |  |  |  |  |
| Female | 1.37***(1.16−1.62) | 1.03 (0.87−1.21) | 1.35**(1.14−1.61) | 1.06 (0.90−1.26) | 1.35**(1.14−1.60) | 1.06 (0.89−1.26) |
| Occupation * Gender |  |  |  |  |  |  |
| professional/white collar * Female | 0.97 (0.79−1.19) | 1.18 (0.96−1.44) | 0.97 (0.79−1.19) | 1.16 (0.95−1.42) | 0.97 (0.79−1.19) | 1.15 (0.94−1.41) |
| Age group (ref: 25 – 34 years) |  |  |  |  |  |  |
| 35 − 44 | 1.51***(1.35−1.68) | 1.36***(1.22−1.52) | 1.46***(1.30−1.65) | 1.43***(1.27−1.62) | 1.46***(1.30−1.65) | 1.45***(1.29−1.64) |
| 45 − 54 | 1.97***(1.67−2.33) | 2.27***(1.93−2.67) | 1.90***(1.59−2.26) | 2.41***(2.03−2.87) | 1.91***(1.60−2.27) | 2.52***(2.12−3.01) |
| Self-rated health | 0.96 (0.89−1.03) | 0.87***(0.81−0.93) | 0.97 (0.90−1.05) | 0.91* (0.84−0.98) | 0.98 (0.91−1.05) | 0.92* (0.85−0.99) |
| Chronic disease (Yes) | 0.96 (0.85−1.09) | 0.95 (0.84−1.07) | 0.97 (0.85−1.10) | 0.95 (0.84−1.07) | 0.96 (0.85−1.10) | 0.90 (0.79−1.02) |
| Disability (Yes) | 0.89 (0.57−1.39) | 0.84 (0.54−1.30) | 0.86 (0.55−1.35) | 0.76 (0.49−1.19) | 0.85 (0.54−1.34) | 0.74 (0.47−1.17) |
| Health behavior |  |  |  |  |  |  |
| Smoking | 0.96 (0.93−1.00) | 0.93***(0.9−0.97) | 0.97 (0.93−1.00) | 0.93**(0.90−0.97) | 0.97 (0.93−1.00) | 0.94**(0.90−0.97) |
| Drinking | 1.03 (0.97−1.08) | 0.99 (0.94−1.04) | 1.03 (0.98−1.08) | 0.99 (0.94−1.04) | 1.03 (0.98−1.08) | 0.99 (0.94−1.04) |
| Exercising | 1.07* (1.01−1.12) | 1.00 (0.94−1.05) | 1.07* (1.01−1.13) | 1.00 (0.95−1.06) | 1.07* (1.02−1.13) | 1.01 (0.96−1.07) |
| Knowledge about vaccines | 1.00 (0.95−1.06) | 0.75***(0.71−0.79) | 1.01 (0.95−1.06) | 0.75***(0.71−0.80) | 1.01 (0.96−1.07) | 0.79***(0.75−0.83) |
| Marital status (ref: single) |  |  |  |  |  |  |
| Married |  |  | 1.02 (0.89−1.18) | 0.87 (0.76−1.00) | 1.02 (0.89−1.17) | 0.85* (0.75−0.98) |
| Divorced |  |  | 0.83 (0.64−1.08) | 0.67**(0.52−0.87) | 0.84 (0.65−1.09) | 0.69**(0.53−0.89) |
| Widowed |  |  | 0.66* (0.47−0.94) | 0.62**(0.44−0.87) | 0.66* (0.46−0.94) | 0.57**(0.40−0.81) |
| Living arrangements (ref: living alone) |  |  |  |  |  |  |
| Living with family members |  |  | 1.18* (1.02−1.37) | 1.03 (0.89−1.18) | 1.18* (1.02−1.37) | 1.08 (0.93−1.25) |
| Co-rental |  |  | 1.04 (0.86−1.26) | 0.95 (0.79−1.14) | 1.04 (0.86−1.26) | 0.96 (0.80−1.16) |
| Else |  |  | 1.17 (0.95−1.44) | 0.98 (0.80−1.21) | 1.17 (0.95−1.44) | 0.97 (0.78−1.19) |
| Family relation |  |  | 0.95 (0.89−1.01) | 0.83***(0.79−0.88) | 0.96 (0.90−1.02) | 0.87***(0.82−0.93) |
| Perceptions of environmental safety |  |  |  |  |  |  |
| Government's prevention and control measures |  |  |  |  | 0.97 (0.90−1.05) | 0.88**(0.82−0.96) |
| Government's prevention and control outcomes |  |  |  |  | 0.99 (0.90−1.08) | 0.73***(0.67−0.79) |
| Concerns about the international outbreak spread |  |  |  |  | 0.99 (0.96−1.03) | 1.09***(1.04−1.13) |

Notes: **p* < 0.05; ***p* < 0.01; ****p* < 0.001

Model 1H: association between occupation and motivation patterns, gender as an intersectional variable, adjusted for age, self-rated health, chronic disease, disability, health behavior, and knowledge about vaccines (individual factors);

Model 2H: association between occupation and motivation patterns, gender as an intersectional variable, adjusted for age, self-rated health, chronic disease, disability, health behavior, and knowledge about vaccines, marital status, living arrangements, and family relation (individual and family factors);

Model 3H: association between occupation and motivation patterns, gender as an intersectional variable, adjusted for age, self-rated health, chronic disease, disability, health behavior, and knowledge about vaccines, marital status, living arrangements, family relation, and perceptions of environmental safety (individual, family and social factors);

S12 Table. Replication of S8 presenting detailed results (Model 1I-3I) on covariates.

| Variable | Model 1I | | Model 2I | | Model 3I | |
| --- | --- | --- | --- | --- | --- | --- |
|  | Motivation patterns (Trust & differential protection) | | Motivation patterns (Trust & differential protection) | | Motivation patterns (Trust & differential protection) | |
|  | Trust & self-protection  OR (95% CI) | Self-protection  OR (95% CI) | Trust & self-protection  OR (95% CI) | Self-protection  OR (95% CI) | Trust & self-protection  OR (95% CI) | Self-protection  OR (95% CI) |
| Migration (ref: non-migrant) |  |  |  |  |  |  |
| Migrant | 0.91 (0.79−1.05) | 0.82**(0.72−0.95) | 0.94 (0.81−1.09) | 0.79**(0.68−0.91) | 0.94 (0.81−1.09) | 0.75***(0.65−0.87) |
| Gender (ref: male) |  |  |  |  |  |  |
| Female | 1.24* (1.05−1.47) | 1.02 (0.86−1.2) | 1.24* (1.05−1.48) | 1.03 (0.87−1.22) | 1.24* (1.04−1.47) | 1.01 (0.85−1.2) |
| Migration * Gender |  |  |  |  |  |  |
| Migrant* Female | 1.13 (0.92−1.39) | 1.16 (0.95−1.43) | 1.12 (0.91−1.38) | 1.20 (0.97−1.47) | 1.12 (0.91−1.38) | 1.22 (0.99−1.5) |
| Age group (ref: 25 – 34 years) |  |  |  |  |  |  |
| 35 − 44 | 1.50***(1.34−1.68) | 1.33***(1.19−1.49) | 1.46***(1.29−1.65) | 1.41***(1.25−1.59) | 1.46***(1.29−1.65) | 1.43***(1.26−1.62) |
| 45 − 54 | 1.94***(1.64−2.3) | 2.18***(1.84−2.57) | 1.90***(1.59−2.26) | 2.33***(1.96−2.78) | 1.90***(1.59−2.27) | 2.42***(2.03−2.89) |
| Self-rated health | 0.96 (0.89−1.03) | 0.87***(0.81−0.94) | 0.97 (0.91−1.05) | 0.92* (0.85−0.99) | 0.98 (0.91−1.05) | 0.93 (0.86−1) |
| Chronic disease (Yes) | 0.96 (0.85−1.09) | 0.95 (0.84−1.08) | 0.97 (0.86−1.1) | 0.95 (0.84−1.08) | 0.97 (0.85−1.1) | 0.90 (0.79−1.02) |
| Disability (Yes) | 0.88 (0.56−1.38) | 0.82 (0.53−1.28) | 0.85 (0.54−1.34) | 0.75 (0.48−1.16) | 0.84 (0.54−1.32) | 0.72 (0.46−1.13) |
| Health behavior |  |  |  |  |  |  |
| Smoking | 0.97 (0.93−1) | 0.94**(0.9−0.97) | 0.97 (0.93−1.01) | 0.94**(0.9−0.98) | 0.97 (0.93−1.01) | 0.94**(0.9−0.98) |
| Drinking | 1.03 (0.97−1.08) | 0.99 (0.94−1.04) | 1.03 (0.98−1.08) | 0.99 (0.94−1.05) | 1.03 (0.98−1.08) | 0.99 (0.94−1.05) |
| Exercising | 1.07* (1.01−1.12) | 0.99 (0.94−1.05) | 1.07* (1.01−1.13) | 1.00 (0.95−1.05) | 1.07* (1.02−1.13) | 1.01 (0.96−1.06) |
| Knowledge about vaccines | 1.00 (0.95−1.06) | 0.74***(0.71−0.79) | 1.01 (0.95−1.06) | 0.75***(0.71−0.8) | 1.01 (0.96−1.07) | 0.79***(0.75−0.83) |
| Marital status (ref: single) |  |  |  |  |  |  |
| Married |  |  | 1.02 (0.89−1.17) | 0.86* (0.75−0.99) | 1.02 (0.89−1.17) | 0.84* (0.73−0.96) |
| Divorced |  |  | 0.83 (0.64−1.08) | 0.66**(0.51−0.86) | 0.84 (0.65−1.09) | 0.67**(0.52−0.87) |
| Widowed |  |  | 0.66* (0.47−0.94) | 0.60**(0.43−0.85) | 0.66* (0.46−0.93) | 0.56**(0.39−0.79) |
| Living arrangements (ref: living alone) |  |  |  |  |  |  |
| Living with family members |  |  | 1.18* (1.02−1.37) | 0.99 (0.86−1.15) | 1.18* (1.02−1.37) | 1.04 (0.9−1.21) |
| Co-rental |  |  | 1.04 (0.86−1.25) | 0.96 (0.8−1.15) | 1.04 (0.86−1.25) | 0.97 (0.81−1.17) |
| Else |  |  | 1.17 (0.95−1.44) | 0.98 (0.8−1.21) | 1.17 (0.95−1.44) | 0.96 (0.78−1.19) |
| Family relation |  |  | 0.95 (0.89−1.01) | 0.83***(0.78−0.88) | 0.95 (0.9−1.01) | 0.87***(0.82−0.92) |
| Perceptions of environmental safety |  |  |  |  |  |  |
| Government's prevention and control measures |  |  |  |  | 0.97 (0.9−1.05) | 0.88**(0.82−0.95) |
| Government's prevention and control outcomes |  |  |  |  | 0.99 (0.9−1.08) | 0.73***(0.67−0.8) |
| Concerns about the international outbreak spread |  |  |  |  | 0.99 (0.96−1.03) | 1.09***(1.04−1.13) |

Notes: **p* < 0.05; ***p* < 0.01; ****p* < 0.001

Model 1I: association between migration and motivation patterns, gender as an intersectional variable, adjusted for age, self-rated health, chronic disease, disability, health behavior, and knowledge about vaccines (individual factors);

Model 2I: association between migration and motivation patterns, gender as an intersectional variable, adjusted for age, self-rated health, chronic disease, disability, health behavior, and knowledge about vaccines, marital status, living arrangements, and family relation (individual and family factors);

Model 3I: association between migration and motivation patterns, gender as an intersectional variable, adjusted for age, self-rated health, chronic disease, disability, health behavior, and knowledge about vaccines, marital status, living arrangements, family relation, and perceptions of environmental safety (individual, family and social factors);

S13 Table. Replication of S8 presenting detailed results (Model 1J-3J) on covariates.

| Variable | Model 1J | | Model 2J | | Model 3J | |
| --- | --- | --- | --- | --- | --- | --- |
|  | Motivation patterns (Trust & differential protection)1 | | Motivation patterns (Trust & differential protection) | | Motivation patterns (Trust & differential protection) | |
|  | Trust & self-protection  OR (95% CI) | Self-protection  OR (95% CI) | Trust & self-protection  OR (95% CI) | Self-protection  OR (95% CI) | Trust & self-protection  OR (95% CI) | Self-protection  OR (95% CI) |
| Region (ref: Chengdu) |  |  |  |  |  |  |
| Shanghai | 1.02 (0.86−1.21) | 1.10 (0.93−1.30) | 1.02 (0.86−1.21) | 1.10 (0.93−1.30) | 1.02 (0.86−1.21) | 1.12 (0.94−1.32) |
| Shenzhen | 0.77**(0.66−0.90) | 0.79**(0.67−0.92) | 0.77**(0.66−0.91) | 0.72***(0.61−0.84) | 0.77**(0.65−0.90) | 0.65***(0.56−0.77) |
| Gender (ref: male) |  |  |  |  |  |  |
| Female | 1.25* (1.05−1.50) | 0.96 (0.80−1.14) | 1.25* (1.05−1.49) | 0.97 (0.81−1.15) | 1.24* (1.04−1.48) | 0.94 (0.78−1.12) |
| Region * Gender |  |  |  |  |  |  |
| Shanghai* Female | 1.04 (0.81−1.34) | 1.47**(1.15−1.89) | 1.05 (0.82−1.35) | 1.47**(1.15−1.88) | 1.05 (0.82−1.35) | 1.50**(1.17−1.93) |
| Shenzhen* Female | 1.07 (0.84−1.37) | 0.90 (0.70−1.15) | 1.07 (0.84−1.37) | 0.96 (0.75−1.24) | 1.07 (0.84−1.37) | 0.96 (0.75−1.24) |
| Age group (ref: 25 – 34 years) |  |  |  |  |  |  |
| 35 − 44 | 1.46***(1.31−1.63) | 1.29***(1.15−1.44) | 1.43***(1.27−1.62) | 1.36***(1.20−1.53) | 1.43***(1.27−1.62) | 1.37***(1.21−1.55) |
| 45 − 54 | 1.84***(1.55−2.18) | 2.05***(1.73−2.42) | 1.80***(1.51−2.15) | 2.17***(1.82−2.59) | 1.81***(1.51−2.16) | 2.23***(1.87−2.66) |
| Self-rated health | 0.97 (0.90−1.04) | 0.87***(0.81−0.94) | 0.98 (0.91−1.06) | 0.92* (0.85−0.99) | 0.99 (0.92−1.06) | 0.93 (0.87−1.01) |
| Chronic disease (Yes) | 0.98 (0.86−1.11) | 0.96 (0.84−1.08) | 0.98 (0.87−1.12) | 0.95 (0.84−1.08) | 0.98 (0.86−1.11) | 0.89 (0.79−1.02) |
| Disability (Yes) | 0.87 (0.56−1.37) | 0.82 (0.53−1.28) | 0.84 (0.54−1.33) | 0.74 (0.47−1.15) | 0.84 (0.53−1.31) | 0.71 (0.45−1.11) |
| Health behavior |  |  |  |  |  |  |
| Smoking | 0.97 (0.93−1.01) | 0.93**(0.90−0.97) | 0.97 (0.93−1.01) | 0.94**(0.90−0.98) | 0.97 (0.93−1.01) | 0.94**(0.90−0.98) |
| Drinking | 1.02 (0.97−1.08) | 0.98 (0.93−1.03) | 1.02 (0.97−1.08) | 0.98 (0.93−1.03) | 1.02 (0.97−1.08) | 0.98 (0.93−1.03) |
| Exercising | 1.06* (1.01−1.12) | 1.00 (0.94−1.05) | 1.07* (1.01−1.12) | 1.00 (0.95−1.06) | 1.07* (1.01−1.13) | 1.01 (0.96−1.07) |
| Knowledge about vaccines | 1.00 (0.95−1.06) | 0.73***(0.69−0.77) | 1.00 (0.95−1.06) | 0.74***(0.70−0.78) | 1.01 (0.95−1.07) | 0.78***(0.74−0.82) |
| Marital status (ref: single) |  |  |  |  |  |  |
| Married |  |  | 1.02 (0.88−1.17) | 0.88 (0.76−1.00) | 1.02 (0.88−1.17) | 0.85* (0.74−0.98) |
| Divorced |  |  | 0.82 (0.63−1.06) | 0.65**(0.50−0.84) | 0.83 (0.64−1.07) | 0.66**(0.51−0.86) |
| Widowed |  |  | 0.69* (0.48−0.98) | 0.65* (0.46−0.92) | 0.68* (0.48−0.97) | 0.60**(0.42−0.86) |
| Living arrangements (ref: living alone) |  |  |  |  |  |  |
| Living with family members |  |  | 1.11 (0.96−1.29) | 0.94 (0.82−1.09) | 1.12 (0.96−1.30) | 0.98 (0.84−1.13) |
| Co-rental |  |  | 0.99 (0.82−1.19) | 0.85 (0.71−1.02) | 0.98 (0.81−1.19) | 0.84 (0.70−1.01) |
| Else |  |  | 1.17 (0.95−1.44) | 0.98 (0.80−1.21) | 1.17 (0.95−1.44) | 0.96 (0.78−1.19) |
| Family relation |  |  | 0.94* (0.88−1.00) | 0.82***(0.77−0.87) | 0.94 (0.89−1.00) | 0.86***(0.81−0.91) |
| Perceptions of environmental safety |  |  |  |  |  |  |
| Government's prevention and control measures |  |  |  |  | 0.97 (0.89−1.05) | 0.88**(0.81−0.95) |
| Government's prevention and control outcomes |  |  |  |  | 0.97 (0.89−1.07) | 0.70***(0.64−0.77) |
| Concerns about the international outbreak spread |  |  |  |  | 1.00 (0.96−1.04) | 1.09***(1.05−1.14) |

Notes: **p* < 0.05; ***p* < 0.01; ****p* < 0.001

Model 1J: association between region and motivation patterns, gender as an intersectional variable, adjusted for age, self-rated health, chronic disease, disability, health behavior, and knowledge about vaccines (individual factors);

Model 2J: association between region and motivation patterns, gender as an intersectional variable, adjusted for age, self-rated health, chronic disease, disability, health behavior, and knowledge about vaccines, marital status, living arrangements, and family relation (individual and family factors);

Model 3J: association between region and motivation patterns, gender as an intersectional variable, adjusted for age, self-rated health, chronic disease, disability, health behavior, and knowledge about vaccines, marital status, living arrangements, family relation, and perceptions of environmental safety (individual, family and social factors);

S14 Table. Sensitivity analyses of SES.

| Variable | Model 1 | | Model 2 | | Model 3 | |
| --- | --- | --- | --- | --- | --- | --- |
|  | Motivation patterns (Trust & differential protection) | | Motivation patterns (Trust & differential protection) | | Motivation patterns (Trust & differential protection) | |
|  | Trust & self-protection  OR (95% CI) | Self-protection  OR (95% CI) | Trust & self-protection  OR (95% CI) | Self-protection  OR (95% CI) | Trust & self-protection  OR (95% CI) | Self-protection  OR (95% CI) |
| Education (ref: illiteracy and primary school) |  |  |  |  |  |  |
| Junior high school | 1.86**(1.18−2.92) | 0.65* (0.44−0.96) | 1.87** (1.19−2.94) | 0.67* (0.45−0.99) | 1.86**(1.18−2.93) | 0.68 (0.46−1.01) |
| Senior high school | 1.74* (1.11−2.75) | 0.51**(0.35−0.76) | 1.76* (1.11−2.78) | 0.53**(0.36−0.79) | 1.76* (1.11−2.77) | 0.56**(0.38−0.83) |
| University and junior college | 1.81* (1.14−2.85) | 0.61* (0.41−0.91) | 1.82* (1.15−2.87) | 0.65* (0.44−0.96) | 1.81* (1.15−2.87) | 0.70 (0.47−1.05) |
| Master and above | 2.03**(1.24−3.32) | 0.86 (0.56−1.32) | 2.06**(1.26−3.39) | 0.90 (0.58−1.38) | 2.07**(1.26−3.39) | 1.01 (0.65−1.56) |
| Income (ref: < 5000, monthly,￥) |  |  |  |  |  |  |
| >=5000 and <10000 | 1.18* (1.03−1.35) | 1.05 (0.92−1.21) | 1.17* (1.02−1.34) | 1.07 (0.94−1.23) | 1.18* (1.03−1.35) | 1.11 (0.97−1.28) |
| >=10000 and <20000 | 1.19* (1.02−1.40) | 1.06 (0.91−1.25) | 1.17 (1.00−1.38) | 1.08 (0.92−1.27) | 1.17 (1.00−1.38) | 1.14 (0.97−1.34) |
| >=20000 and <50000 | 1.19* (1.00−1.42) | 1.10 (0.92−1.31) | 1.18 (0.99−1.40) | 1.12 (0.94−1.33) | 1.18 (0.99−1.40) | 1.17 (0.98−1.40) |
| >=50000 | 1.21* (1.01−1.44) | 1.13 (0.95−1.34) | 1.20* (1.01−1.43) | 1.17 (0.99−1.40) | 1.20* (1.01−1.43) | 1.25* (1.05−1.49) |
| Occupation (ref: manual/unskilled service) |  |  |  |  |  |  |
| professional/white collar | 0.95 (0.85−1.07) | 1.00 (0.90−1.12) | 0.95 (0.85−1.06) | 0.99 (0.89−1.11) | 0.95 (0.85−1.07) | 1.02 (0.91−1.14) |
| Age group (ref: 25 – 34 years) |  |  |  |  |  |  |
| 35 − 44 | 1.51***(1.35−1.69) | 1.35***(1.20−1.51) | 1.47***(1.30−1.65) | 1.42***(1.25−1.60) | 1.47***(1.30−1.65) | 1.44***(1.28−1.63) |
| 45 − 54 | 2.05***(1.73−2.42) | 2.2***(1.86−2.60) | 1.98***(1.65−2.36) | 2.35***(1.97−2.81) | 1.99***(1.66−2.38) | 2.52***(2.10−3.01) |
| Gender (ref: male) |  |  |  |  |  |  |
| Female | 1.35***(1.20−1.52) | 1.12 (0.99−1.26) | 1.34***(1.18−1.51) | 1.15* (1.02−1.30) | 1.34***(1.18−1.51) | 1.15* (1.01−1.30) |
| Self-rated health | 0.96 (0.90−1.04) | 0.87***(0.81−0.93) | 0.98 (0.91−1.05) | 0.91* (0.84−0.98) | 0.98 (0.91−1.06) | 0.93 (0.86−1.00) |
| Chronic disease (Yes) | 0.98 (0.86−1.11) | 0.96 (0.85−1.09) | 0.99 (0.87−1.12) | 0.96 (0.85−1.09) | 0.98 (0.86−1.11) | 0.91 (0.81−1.04) |
| Disability (Yes) | 0.93 (0.59−1.46) | 0.83 (0.53−1.29) | 0.90 (0.57−1.41) | 0.76 (0.49−1.19) | 0.88 (0.56−1.38) | 0.75 (0.48−1.19) |
| Health behavior |  |  |  |  |  |  |
| Smoking | 0.97 (0.93−1.01) | 0.94**(0.91−0.98) | 0.97 (0.93−1.01) | 0.95**(0.91−0.99) | 0.97 (0.93−1.01) | 0.95* (0.92−0.99) |
| Drinking | 1.02 (0.97−1.08) | 0.99 (0.94−1.04) | 1.02 (0.97−1.08) | 0.99 (0.94−1.04) | 1.02 (0.97−1.08) | 0.98 (0.93−1.04) |
| Exercising | 1.06* (1.00−1.12) | 0.99 (0.94−1.05) | 1.06* (1.01−1.12) | 1.00 (0.94−1.05) | 1.06* (1.01−1.12) | 1.00 (0.95−1.06) |
| Knowledge about vaccines | 1.00 (0.94−1.05) | 0.75***(0.71−0.79) | 1.00 (0.95−1.06) | 0.75***(0.71−0.80) | 1.01 (0.95−1.06) | 0.79***(0.74−0.83) |
| Marital status (ref: single) |  |  |  |  |  |  |
| Married |  |  | 1.03 (0.90−1.18) | 0.89 (0.77−1.02) | 1.03 (0.90−1.18) | 0.87 (0.76−1.00) |
| Divorced |  |  | 0.85 (0.65−1.1) | 0.69**(0.53−0.89) | 0.85 (0.66−1.11) | 0.71* (0.54−0.92) |
| Widowed |  |  | 0.68* (0.48−0.96) | 0.62**(0.44−0.88) | 0.67* (0.47−0.96) | 0.59**(0.42−0.84) |
| Living arrangements (ref: living alone) |  |  |  |  |  |  |
| Living with family members |  |  | 1.15 (1.00−1.34) | 1.01 (0.87−1.16) | 1.16 (1.00−1.34) | 1.04 (0.90−1.21) |
| Co-rental |  |  | 1.04 (0.86−1.25) | 0.95 (0.79−1.14) | 1.04 (0.86−1.25) | 0.95 (0.79−1.15) |
| Else |  |  | 1.18 (0.95−1.45) | 0.98 (0.79−1.20) | 1.18 (0.95−1.46) | 0.97 (0.79−1.20) |
| Family relation |  |  | 0.94* (0.88−1.00) | 0.83***(0.78−0.88) | 0.95 (0.89−1.01) | 0.86***(0.81−0.92) |
| Perceptions of environmental safety |  |  |  |  |  |  |
| Government's prevention and control measures |  |  |  |  | 0.97 (0.90−1.05) | 0.88**(0.82−0.95) |
| Government's prevention and control outcomes |  |  |  |  | 0.98 (0.89−1.07) | 0.72***(0.66−0.79) |
| Concerns about the international outbreak spread |  |  |  |  | 1.00 (0.96−1.04) | 1.09***(1.05−1.14) |

Notes: **p* < 0.05; ***p* < 0.01; ****p* < 0.001

Model 1: association between education, income, occupation and motivation patterns, adjusted for age, gender, self-rated health, chronic disease, disability, health behavior, and knowledge about vaccines (individual factors);

Model 2: association between education, income, occupation and motivation patterns, adjusted for age, gender, self-rated health, chronic disease, disability, health behavior, and knowledge about vaccines, marital status, living arrangements, and family relation (individual and family factors);

Model 3: association between education, income, occupation and motivation patterns, adjusted for age, gender, self-rated health, chronic disease, disability, health behavior, and knowledge about vaccines, marital status, living arrangements, family relation, and perceptions of environmental safety (individual, family and social factors);
