# Supplementary material for: An exploration on COVID-19 vaccination motivation patterns from the perspective of the Chaxu culture in metropolis of China: A multi-center study
Source: Front Public Health. 2022 Dec 22;10:1065043. doi: 10.3389/fpubh.2022.1065043 (PMC9815457; doi:10.3389/fpubh.2022.1065043)
Supplement: Supplementary file 2 [file Data_Sheet_2.docx]

**Additional File 1. Measures**

**COVID-19 vaccination motivations**

What are your COVID-19 vaccination motivations? (multiple choice question)

1. Trust in the safety of vaccines
2. Trust in the effectiveness of vaccines
3. Self-protection
4. Protection of family members
5. Protection of neighbors, friends and colleagues
6. Contributing to herd immunity
7. Perceived personal obligations influenced by media publicity
8. People around have vaccinated
9. Vaccinated people around me did not experience side effects
10. Company-based mobilization
11. Community mobilization

**Other measures**

*Individual factors*

1. How do you feel about your overall health, looking at the recent seven days? (self-rated health)

1 = very poor

2 = poor

3 = fair

4 = good

5 = very good

1. Do you have any chronic diseases diagnosed by doctors? (chronic diseases)

0 = No

1 = Yes

1. Do you have any disability? (disability)

0 = No

1 = Yes

1. Do you smoke now? (smoking)

1 = never

2 = basically not

3 = occasionally

4 = sometimes

5 = often

1. Do you drink now? (drinking)

1 = never

2 = basically not

3 = occasionally

4 = sometimes

5 = often

1. Do you exercise now? (exercise)

1 = never

2 = basically not

3 = occasionally

4 = sometimes

5 = often

1. Do you know about Covid-19 vaccination (such as the adverse reactions and contraindications)?

1 = unfamiliar very much

2 = unfamiliar

3 = moderate

4 = familiar

5 = familiar very much

*Family factors*

1. Which statement best describes your present living arrangements?

1 = living alone

2 =living with family members

3 = co-rental

4 = else

1. Which statement best describes your family relation?

1 = disharmonious very much

2 = disharmonious

3 = fair

4 = harmonious

5 = harmonious very much

*Social factors*

1. What do you think of the government's prevention and control measures?

1 = very poor

2 = poor

3 = fair

4 = good

5 = very good

2. What do you think of the government's prevention and control outcomes?

1 = very poor

2 = poor

3 = fair

4 = good

5 = very good

3. Do you concern that the spread of the international epidemic has hidden dangers for your safety?

1 = very much

2 = much

3 = moderate

4 = seldom

5 = not at all
